# Gastric cancer cell death analyzed by live cell imaging of spheroids

**DOI:** 10.1038/s41598-022-05426-1

**Published:** 2022-01-27

**Authors:** George Alzeeb, Danielle Arzur, Valérie Trichet, Matthieu Talagas, Laurent Corcos, Catherine Le Jossic-Corcos

**Affiliations:** 1 Univ Brest, Inserm, EFS, UMR 1078, GGB, 29200 Brest, France; 2 Inserm, Univ Nantes, UMR 1238 Phy-Os “Bone Sarcomas and Remodeling of Calcified Tissues”, 44035 Nantes, France; 3 Univ Brest, Laboratoire Interactions Epithéliums-Neurones, 29200 Brest, France; 4 Department of Dermatology, University Hospital of Brest, 29200 Brest, France; 5 Inserm, Univ Brest, EFS, UMR 1078, GGB, 29200 Brest, France; 6 CHU de Brest, Inserm, Univ Brest, EFS, UMR 1078, GGB, 29200 Brest, France

**Keywords:** Biological models, Gastrointestinal cancer, Cancer, Cancer therapy, Chemotherapy

## Abstract

Gastric cancer (GC) is the third cause of cancer-related mortality worldwide and is often diagnosed at advanced stages of the disease. This makes the development of more comprehensive models and efficient treatments crucial. One option is based on repurposing already marketed drugs as adjuvants to chemotherapy. Accordingly, we have previously developed the combination of docetaxel and the cholesterol-lowering drug, lovastatin, as a powerful trigger of HGT-1 human GC cells’ apoptosis using 2D cultures. Because 3D models, known as spheroids, are getting recognized as possibly better suited than 2Ds in toxicological research, we aimed to investigate the efficacy of this drug combination with such a model. We established monocellular spheroids from two human (GC) cell lines, HGT-1 and AGS, and bicellular spheroids from these cells mixed with cancer-associated fibroblasts. With these, we surveyed drug-induced cytotoxicity with MTT assays. In addition, we used the Incucyte live imaging and analysis system to follow spheroid growth and apoptosis. Taken together, our results showed that the lovastatin + docetaxel combination was an efficient strategy to eliminate GC cells grown in 2D or 3D cultures, lending further support in favor of repurposing lovastatin as an adjuvant to taxane-based anticancer treatment.

## Introduction

With over one million new cases annually, gastric cancer (GC) is the fifth most diagnosed malignancy. The high mortality rate makes it the fourth most common cause of cancer-related deaths, with 768,793 deaths worldwide in 2018^1^. There are marked geographical differences in the incidence of GC. It is highest in Central and East Asia (71% of cases) followed by Eastern Europe (10%) and Latin America (6%)^2^.

GC is difficult to detect at an early stage, in absence of highly specific warning signs, and roughly 30% of patients have metastases at diagnosis, which results in poor prognosis^3^. Surgical resection is the main treatment for localized forms. Nonetheless, complementary approaches, such as adjuvant chemotherapy, have shown better survival rates^4,5^. However, for advanced GC, improving treatment efficacy remains a major challenge and calls for an urgent need to develop innovative therapies^6^. Inhibition of programmed death-1 (PD-1)/programmed death-ligand 1 (PD-L1) axis with immune checkpoint inhibitors (ICI) has recently been emerging as a novel treatment strategy for advanced GC^7^. Combined treatment is often the basis of current chemotherapy regimen, by which anti-tumor agents show stronger effects^8^. This may extend to drug repurposing as an alternative strategy^9^.

Statins are widely used for the treatment of hypercholesterolemia^10^. Lovastatin, like other statins, reduces serum cholesterol levels through inhibition of the 3-hydroxy-3-methyl-glutaryl-coenzyme A reductase (HMG-CoA Red), a rate-limiting enzyme in the mevalonate pathway^11^. In addition, a large amount of in vitro and in vivo data have suggested that statins bear anti-tumor activity potential, since they decreased cellular proliferation, inhibited metastasis and induced apoptosis^12–16^ including in human breast cancer^17^, esophageal carcinoma^18^, melanoma^19^ cells, or breast cancer stem cells^20^. The synergism between lovastatin and chemotherapeutic agents, such as doxorubicin and idarubicin, has also been reported in different cancers^21–23^.

Two-dimensional (2D) cell cultures have largely contributed to the development of many cancer therapies. However, the types of cell–cell and cell–matrix interactions generally dictated, for epithelial cells, by the necessity to adhere to a solid support, may make 2D models sub-optimal in absence of micro-environmental constraints and dynamic interactions as they occur in tumors^24^. To better mimic the functional aspects of tissues and present a more realistic model of biological responses, three-dimensional (3D) culture systems have been increasingly recognized as more reliable in vitro test models^25^. Multicellular tumor spheroids (MCTS), which associate one or more cell types, are one of the most extensively explored models in preclinical oncology research^26^. The potential of MCTS in predicting the in vivo efficacy of different chemotherapeutic agents has been clearly evidenced, and the responses to treatment in the MCTS model could be closer to the in vivo situation^27,28^. Indeed, various factors such as cell–cell interactions, metabolic status and expression of drug-resistance genes may be different between 2D and 3D cultures and could affect evaluation of drug activity^29^. An important characteristic of solid tumor microenvironments is their heterogeneous cellular composition^30^. The cross-talk between cancer cells and stroma components, such as fibroblasts, endothelial and immune cells, influences various features related to tumor progression or cell invasion^31,32^. The model of hetero-type MCTS overcomes some of the limitations of 2D co-cultures and provides a closer resemblance to tumors^33^.

Several spheroid engineering methodologies have been developed, following the pioneering studies by Sutherland et al. who established MCTS in the 1970s^34^. Novel technologies combining round-bottom geometry with ultra-low attachment (ULA) surface chemistry allowed standardizing 3D cultures and generated reproducible MCTS, which qualifies this model for medium–high throughput screening of anticancer drugs^35^. Recently, assays that measure the effects of drugs in real-time have been designed to mimic in vivo drug responses^36,37^. Medium–high throughput imaging systems, such as Incucyte (Sartorius, Essen Bioscience), are well-suited for this purpose, as they enable the long term follow-up of growth or death of 3D cultures^38^.

In a previous study, our team reported that the association of lovastatin and docetaxel, an anticancer taxane compound, which affects microtubule dynamics^39,40^ and is used to treat GC and other solid tumors^41^, provided an over-additive apoptotic response of the human HGT-1 GC cell line grown in standard 2D conditions^42^. Since the 3D model has been recognized as often more resistant to cytotoxic drugs than the 2D model^43,44^, we decided to ask if our previous observations that demonstrated the potential benefit of associating lovastatin + docetaxel as a strong trigger of cancer cell death, would still hold for two human GC cell lines, HGT-1 and AGS, grown in 3D. To this end, we established a spheroid model, either as a single cell type, or combined with cancer-associated fibroblasts (CAF). We made use of the Incucyte live imaging and analysis system to determine the growth and the cytotoxicity or apoptosis inducing potential of lovastatin and docetaxel.

## Results

### Docetaxel and lovastatin are cytotoxic to human gastric cancer HGT-1 and AGS cells in 2D model

**Figure 1:**
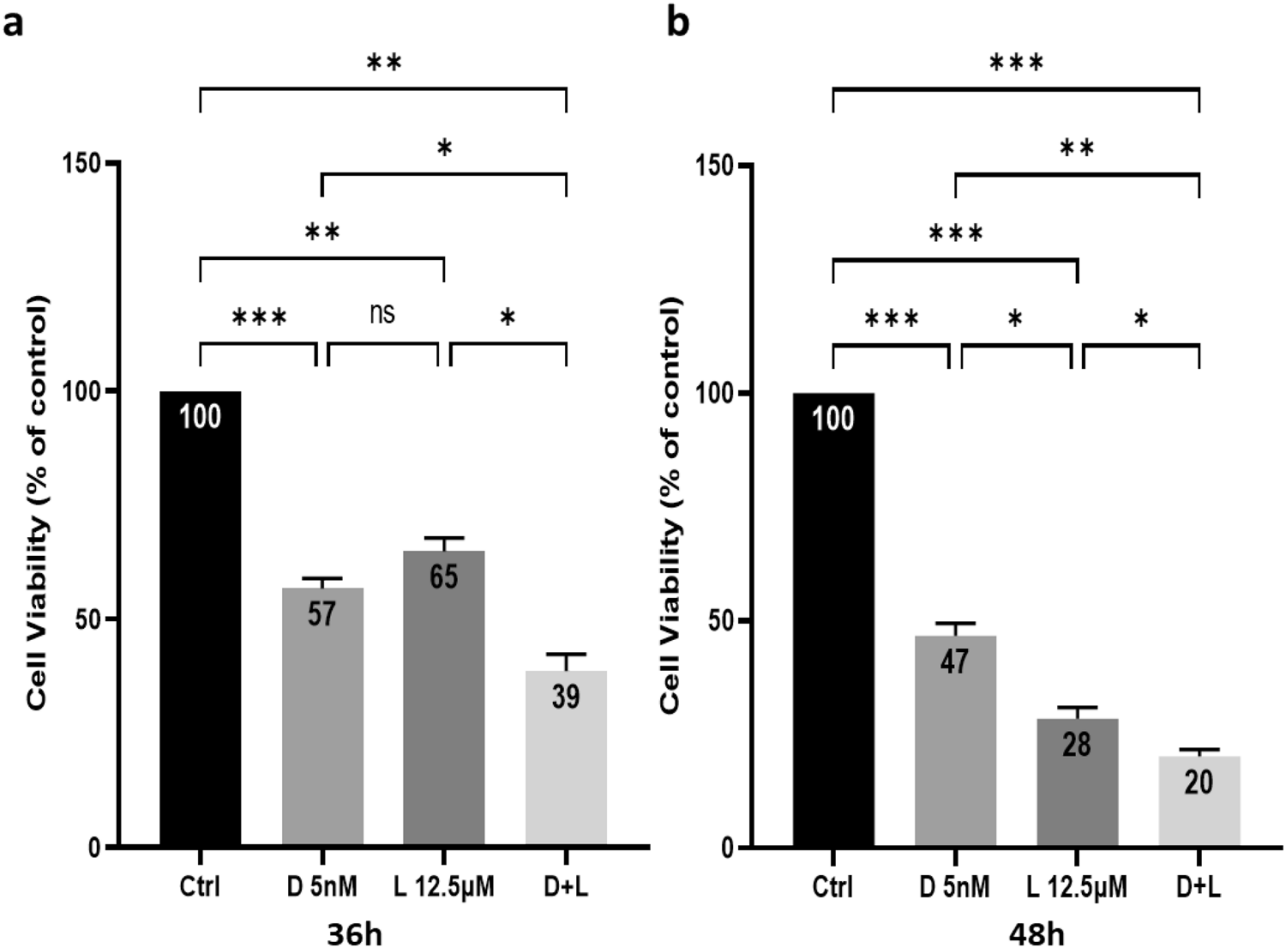
Toxicity of docetaxel and lovastatin in human gastric cancer HGT-1 cells in 2D culture. The cells were incubated at 37 °C for 36 (**a**) and 48 (**b**) h of treatment with 5 nM docetaxel (D 5 nM), 12.5 µM lovastatin (L 12.5 µM) and 5 nM docetaxel + 12.5 µM lovastatin (D + L). Cell viability was determined by the MTT assay. The results are shown as the mean ± SD of n = 3 independent experiments with four technical replicates in each. ns, *p* > 0.05; *p ≤ 0.05; **p ≤ 0.01; ***p ≤ 0.001, one-way ANOVA followed by Tukey analysis.

We have shown previously that lovastatin enhanced the apoptosis induction brought about by docetaxel in HGT-1 human gastric cancer cells in 2D^42^. In the present study, we selected, for further in vitro experiments, the same concentrations of 5 nM docetaxel (D 5 nM), 12.5 µM lovastatin (L 12.5 µM) or a combination of 5 nM docetaxel + 12.5 µM lovastatin (D + L). To evaluate the cytotoxicity of the drugs, 2D cultured HGT-1 cells were treated with D 5 nM, L 12.5 µM and D + L for 36 h (Fig. 1a) and 48 h (Fig. 1b), and cytotoxicity was determined using MTT assays. The results showed a significant reduction of cell viability down to 57% (*p* < 0.001) and 65% (*p* < 0.01), respectively, by D 5 nM and L 12.5 µM treatments at 36 h, and down to 47% (*p* < 0.001) and 28% (*p* < 0.001) after 48 h of treatment. The exposure to both drugs had a cumulative effect on cell viability that was reduced down to 39% (*p* < 0.001) for 36 h of treatment and to 20% (*p* < 0.001) for 48 h. We have also evaluated the cytotoxicity effect of D 5 nM and / or L 12.5 µM of AGS cells after 48 h of treatment. Similar results were obtained for AGS cells (Supplementary Fig. 1).

### HGT-1 and AGS gastric cancer cells efficiently develop as MCTS

**Figure 2:**
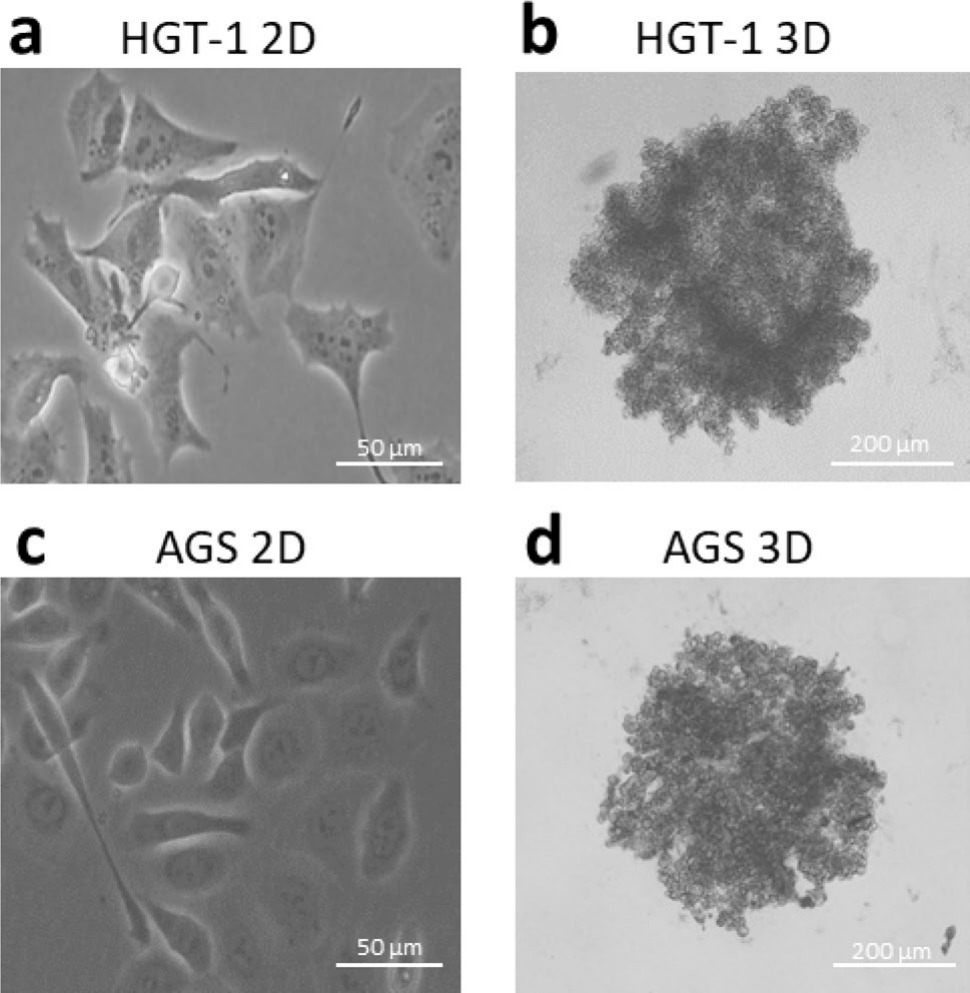
Phase contrast and bright field photographs of HGT-1 and AGS cells in 2D or 3D cultures. Bright field photographs of HGT-1 (**a**: 2D, **b**: 3D-after 6 days of culture) and AGS (**c**: 2D, **d**: 3D-after 6 days of culture) human GC cell lines.

The non-adherent surface method has been widely used in GC studies for its advantage to allow production of spheroids of uniform sizes, an essential requirement for cytotoxicity screening. HGT-1 cells were seeded (500 cells/well) into 96-well ultra-low attachment plates and cultured under standard conditions to develop into spheroids. MCTS formation was rapid and highly reproducible using the HGT-1 cell line as well as with AGS cells (Fig. 2) grown in ultra-low attachment dishes. AGS spheroids were more compact than HGT-1 spheroids. AGS spheroids, but not HGT-1 spheroids showed a few necrotic cells in the central areas as expected from compact spheroid structures. A few mitotic figures were visible on spheroid sections (Supplementary Fig. 2). To get equal sizes of 6 days-old spheroids, we generated AGS spheroids by seeding 1000 cells/well, instead of 500 for HGT-1 cells. Incubation for 6 days alone and without matrix was sufficient to form tightly packed spheroids. Six days-old HGT-1 spheroids were uniform with a diameter of about 540 ± 45 µm and about 6.5 ± 0.2 × 10^3^ cells each. Six days-old AGS spheroids were in the same size range with about 14 ± 0.3 × 10^3^ cells each.

### Docetaxel and lovastatin strongly reduce HGT-1 MCTS growth

**Figure 3:**
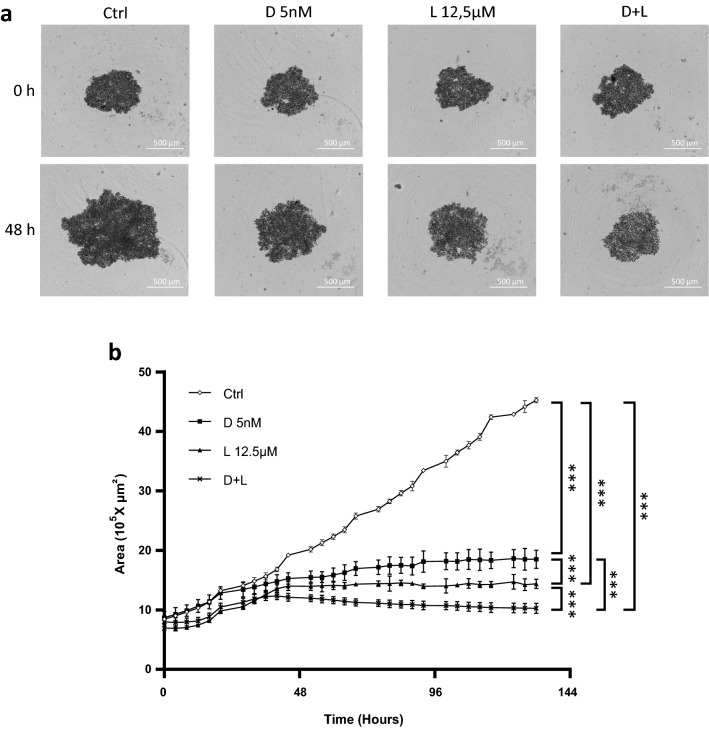
Effects of docetaxel and lovastatin treatments on the growth of HGT-1 spheroids. (**a**) Bright field photographs of 6 days-old HGT-1 MCTS at 0 h and 48 h of treatment with 5 nM docetaxel (D 5 nM), 12.5 µM lovastatin (L 12.5 µM) and 5 nM docetaxel + 12.5 µM lovastatin (D + L), captured with the Incucyte live imaging. (**b**) Real-time growth monitoring (Area 10^5^ × µm^2^) of HGT-1 MCTS was performed using the Incucyte live imaging and analysis system. Six days-old spheroids (starting as 0 on the graph) were treated with 5 nM docetaxel (D 5 nM) (black squares), 12.5 µM lovastatin (L 12.5 µM) (black triangles) or 5 nM docetaxel + 12.5 µM lovastatin (D + L) (black X) for up to 144 h. Control (Ctrl) MCTS are shown as white lozenges. The results are shown as the mean ± SD of n = 3 independent experiments with four technical replicates in each. ***p ≤ 0.001, one-way ANOVA followed by Tukey analysis.

To characterize the effects of the drugs on the growth of HGT-1 MCTS, we assessed their effects in real-time. Six days-old HGT-1 spheroids were treated with D 5 nM, L 12.5 µM and D + L. Spheroid sizes, recorded using the Incucyte live imaging and analysis system, showed that D 5 nM, L 12.5 µM and D + L induced reduction in spheroid growth after 48 h, as shown by bright field photographs of spheroids in Fig. 3a. The effects of the drugs on growth reduction of HGT-1 spheroids, were analyzed in real-time after up to 144 h. The most effective inhibition of growth was achieved using both drugs (Fig. 3b). Hence, real-time area monitoring showed that docetaxel reduced the size of GC MCTS, and this effect was strongly enhanced by lovastatin.

### Docetaxel and lovastatin trigger strong cytotoxicity in HGT-1 and AGS MCTS

**Figure 4:**
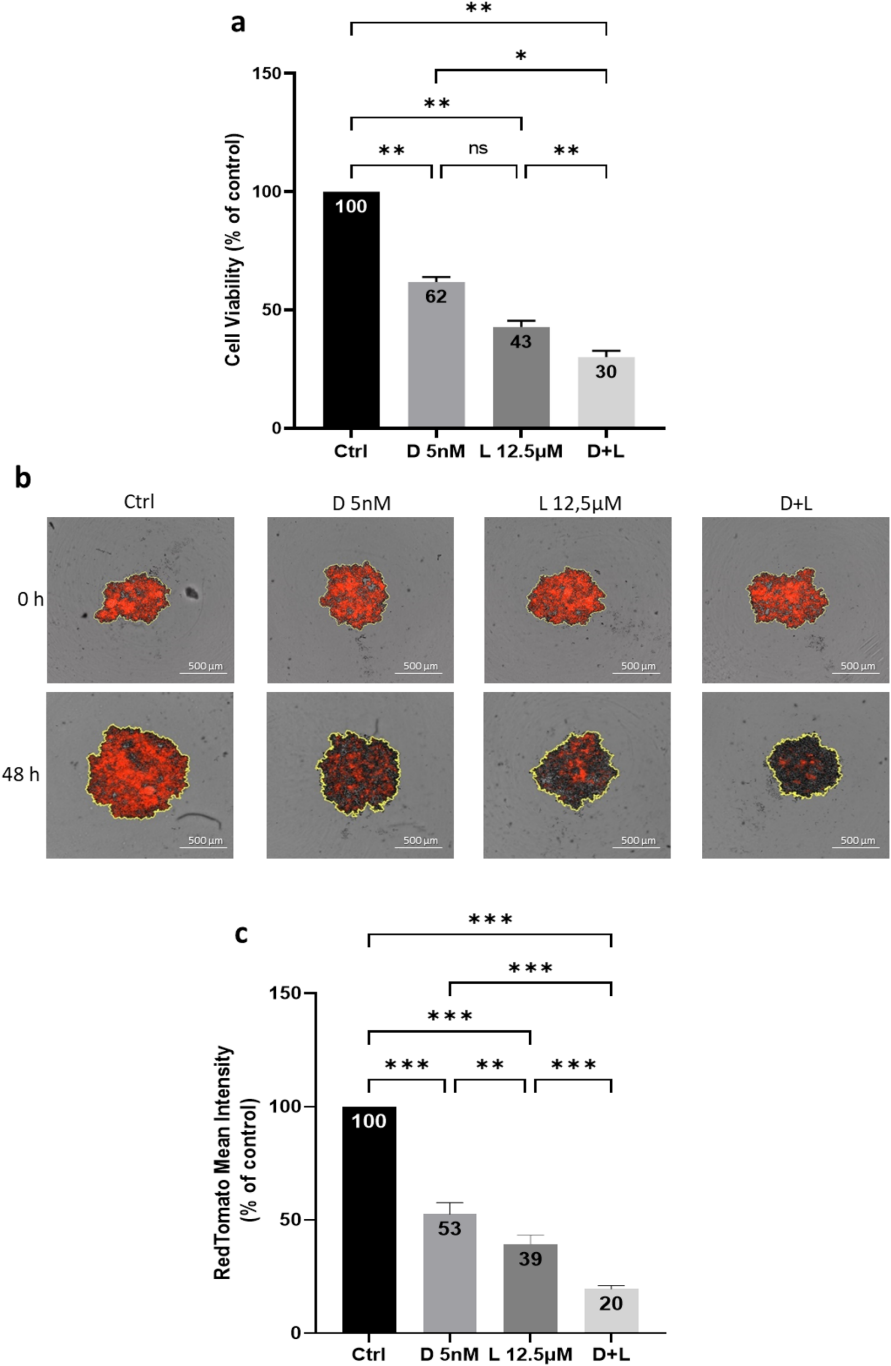
Cytotoxicity of docetaxel and lovastatin for HGT-1 MCTS. (**a**) Cell viability was determined by the MTT assay after 48 h of treatment with 5 nM docetaxel (D 5 nM), 12.5 µM lovastatin (L 12.5 µM) and 5 nM docetaxel + 12.5 µM lovastatin (D + L) of six days-old HGT-1 spheroids. The results are shown as the mean ± SD of n = 3 independent experiments with four technical replicates in each. ns, *p* > 0.05; *p ≤ 0.05; **p ≤ 0.01, one-way ANOVA followed by Tukey analysis. (**b**) Red fluorescence photographs of 6 days-old spheroids formed by HGT-1cells expressing RedTomato at 0 h and 48 h of treatment with D 5 nM, L 12.5 µM and D + L captured with the Incucyte live imaging. (**c**) Fluorescence signal generation by 6 days-old HGT-1-RedTomato MCTS exposed to 5 nM docetaxel (D 5 nM), 12.5 µM lovastatin (L 12.5 µM) and 5 nM docetaxel + 12.5 µM lovastatin (D + L) for 48 h. The results are shown as the mean ± SD of n = 3 independent experiments with four technical replicates in each. **p ≤ 0.01; ***p ≤ 0.001, one-way ANOVA followed by Tukey analysis.

To examine the cell killing effects of docetaxel and lovastatin in a 3D culture system of GC cells, we performed MTT assays, with some modifications compared to the standard assay (see the “Materials and Methods” section). Six days-old HGT-1 spheroids were treated for 48 h by D 5 nM, L 12.5 µM and D + L before adding the MTT reagent. As compared to control, treatment with D 5 nM or L 12.5 µM resulted in reduction of MCTS viability down to 62% (*p* < 0.01) and 43% (*p* < 0.01) of control, respectively. The association of D 5 nM and L 12.5 µM showed the strongest cytotoxicity against HGT-1 MCTS and reduced cells viability down to 30% (*p* < 0.01) (Fig. 4a). Dynamic recording of the effect of drugs shows that control, untreated spheroids kept growing over time, whereas treated spheroids stopped growing and eventually regressed (Supplementary Videos, cytotoxicity files). These results indicated that docetaxel and lovastatin possessed strong cell killing activity in 3D spheroids of HGT-1 cells, similarly to 2D-grown cells (Fig. 1). These results were correlated with MCTS HGT-1 growth, where all treatments reduced spheroid sizes as shown in Fig. 3. We have also examined the cell killing effects of D 5 nM, L 12.5 µM and D + L on 6 days-old AGS MCTS using the same MTT assay. Similar results were obtained 48 h after treatment for 3D-grown AGS cells (Supplementary Fig. 3).

In order to appreciate the effects of the drugs by other means, we generated an HGT-1 sub-population labeled with a RedTomato tag. Such cells should allow for a simplified measurement of cytotoxicity since dead cells are expected to lose RedTomato expression, allowing direct reading of the drop in RedTomato fluorescence intensity by Incucyte as an indicator of decreased cell viability, as reported in the case of GFP protein drop in cells dying in response to drugs^45^. HGT-1 and HGT-1-RedTomato cells were equally sensitive to the 2 cytotoxic drugs in monolayer cultures (Supplementary Fig. 4). Red fluorescence photographs of 6 days-old spheroids treated by D 5 nM, L 12.5 µM and D + L at 0 and 48 h are shown in Fig. 4b. Direct measurement of RedTomato mean intensity of 6 days-old spheroids after exposure to D 5 nM and L 12.5 µM for 48 h produced results that overlapped well with those obtained in the MTT assay. While D 5 nM and L 12.5 µM reduced fluorescence intensity down to 53% (*p* < 0.001) and 39% (*p* < 0.001), respectively, the strongest effects were obtained upon addition of both compounds (20%, *p* < 0.001) (Fig. 4c).

### Docetaxel and lovastatin trigger HGT-1 and AGS spheroid cells apoptosis

**Figure 5:**
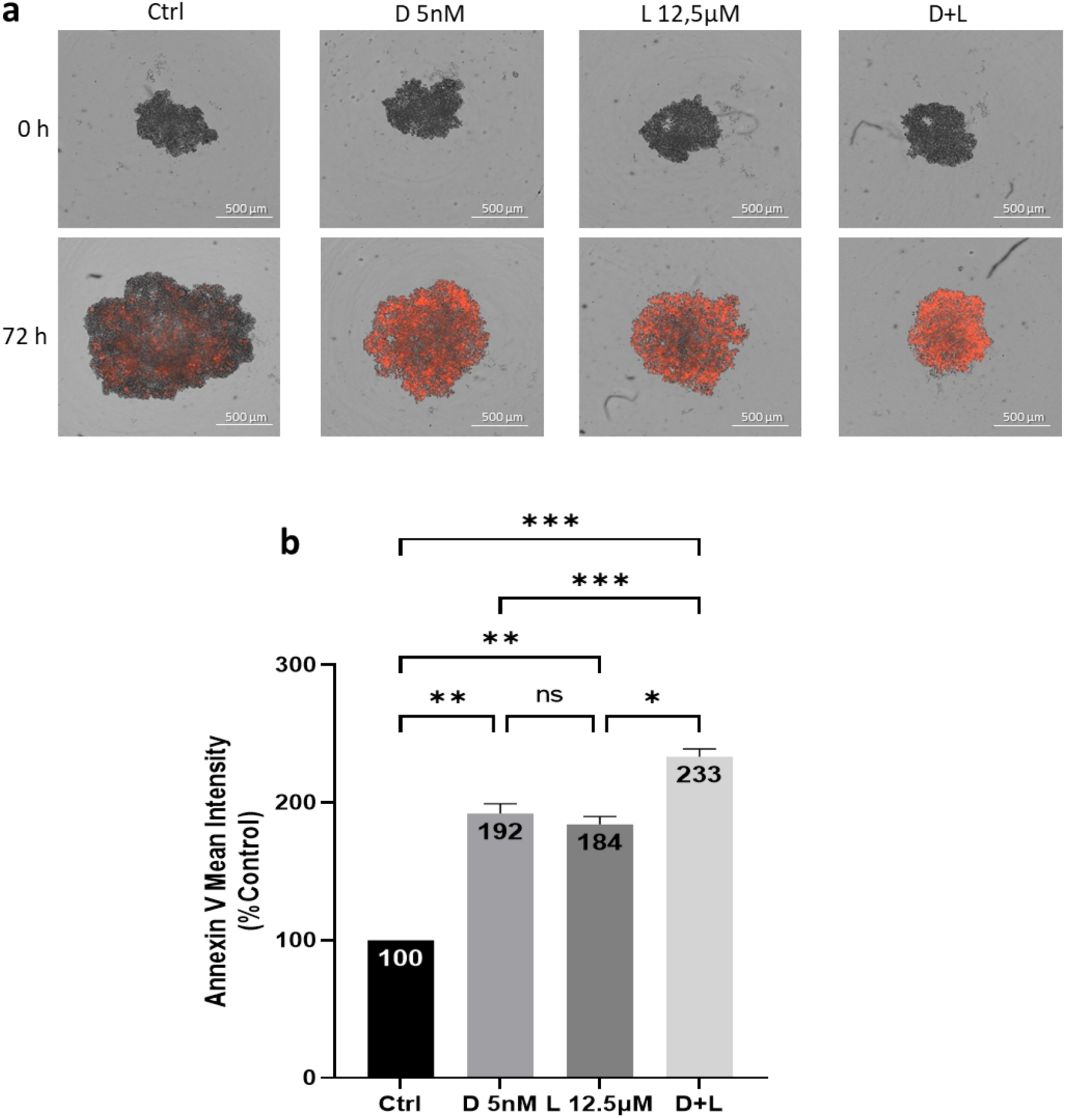
Apoptosis induction by docetaxel and lovastatin in HGT-1 spheroids. (**a**) Red fluorescence photographs of HGT-1 spheroids at 0 h and 72 h of treatment with 5 nM docetaxel (D 5 nM), 12.5 µM lovastatin (L 12.5 µM) and 5 nM docetaxel + 12.5 µM lovastatin (D + L) in presence of Annexin V fluorescent reagent (Incucyte) captured with the Incucyte live imaging. (**b**) Annexin V binding on 6 days-old HGT-1 MCTS after 72 h of treatment with 5 nM docetaxel (D 5 nM), 12.5 µM lovastatin (L 12.5 µM) and 5 nM docetaxel + 12.5 µM lovastatin (D + L). The Annexin V reagent was added at the same time as the drugs. The results are shown as the mean ± SD of n = 3 independent experiments with four technical replicates in each. ns, *p* > 0.05; *p ≤ 0.05; **p ≤ 0.01; ***p ≤ 0.001, one-way ANOVA followed by Tukey analysis.

To further examine if docetaxel and lovastatin-induced cell viability reduction was related to apoptosis, we performed Annexin V staining followed by fluorescence-activated cell intensity measurement using Incucyte. Red fluorescence photographs of 6 days-old HGT-1 spheroids showing binding of Annexin V at 0 and 72 h of treatment with D 5 nM, L 12.5 µM, and D + L are shown in Fig. 5a. Concurrently with the reduced cell viability, apoptosis increased dramatically in response to the drugs. As shown in Fig. 5b, Annexin V fluorescence intensity was increased in response to D 5 nM (by 192%, *p* < 0.01) and L 12.5 µM (by 184%, *p* < 0.01) after 72 h. In addition, the highest apoptosis level (233%, *p* < 0.001) was obtained after exposure to both drugs (D + L). To characterize, in more details the effects of the drugs on apoptosis induction of HGT-1 MCTS, we assessed their effects in real-time (Supplementary Fig. 5) and we determined the level of nuclei fragmentation by Hoechst 33342 staining of the cells (Supplementary Fig. 6a). Comparable results were obtained for AGS cells (Supplementary Fig. 6b). In addition, caspases 3 and 7 activity was increased by two-fold in both HGT-1 and AGS spheroids (Supplementary Fig. 6c,d). Altogether, these results showed that combined treatment promoted high levels of apoptosis in human HGT-1 GC spheroids, similarly to 2D monolayers^42^, as well as in AGS cells.

### Bi-cellular MCTS show sensitivity to docetaxel and lovastatin

**Figure 6:**
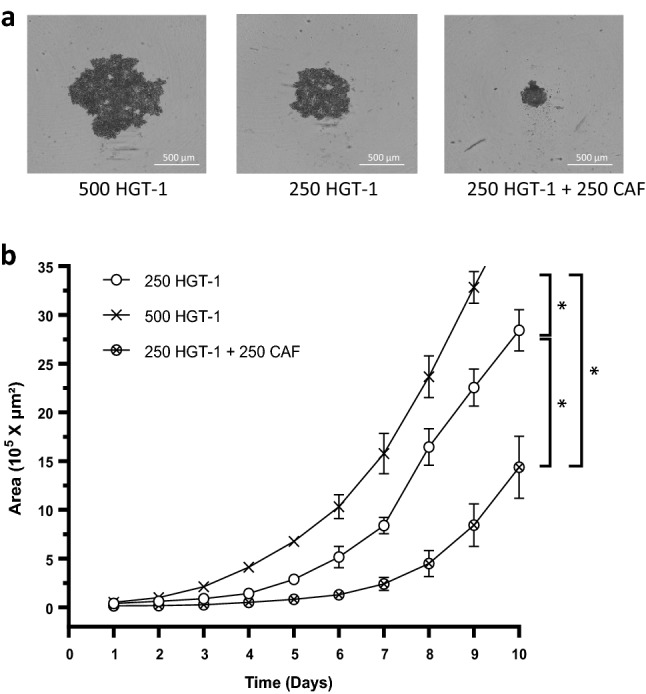
Growth of bicellular MCTS. (**a**) Bright field photographs of 6 days-old monocellular spheroids formed from 500 or 250 HGT-1 cells and bi-cellular (250 CAF + 250 HGT-1) spheroids captured with the Incucyte live imaging. (**b**) Real-time growth monitoring (Area 10^5^ × µm^2^) was performed using the Incucyte live imaging. Monocellular MCTS generated from: (i) 250 HGT-1 cells is represented by white circles, (ii) 500 HGT-1 cells is represented by black X symbol and (iii) bicellular MCTS generated from 250 HGT-1 + 250 CAF is represented by X inside circles. The results are shown as the mean ± SD of n = 3 independent experiments with four technical replicates in each. *p ≤ 0.05; one-way ANOVA followed by Tukey analysis.

In order to better mimic tumors, we established hetero-type MCTS from human HGT-1 or AGS GC cells and human CAF in primary culture. We explored the effects of different ratio of GC cells to CAF: 1:1; 1:2; 1:5; 1:9 on growth. In addition, we followed drug response, as determined by spheroid growth for the 1:1; 1:2 and 1:5 ratios. We observed that the effect of drugs was similar between all ratio conditions. We may hypothesize that CAF did not strongly influence the response of the spheroids to lovastatin and/or docetaxel under our conditions. Therefore, we fixed the ratio at 1:1. Gene expression analysis showed that the CAF grown in 2D confirmed their stromal nature, as shown for αSMA, FAP, VIM, FGF-2, IL-6 and SDF-1 markers^46^, while these genes were essentially not expressed in HGT-1 or AGS cells (Supplementary Fig. 7). Bright field photographs of 6 days-old mono-cellular HGT-1 spheroids (500 and 250 cells) and 250 HGT-1 + 250 CAF bi-cellular spheroids are shown in Fig. 6a. MCTS growth and shape were analyzed over time (Fig. 6b). Bi-cellular (250 CAF + 250 HGT-1) spheroids were more compact compared to HGT-1 spheroids; they reached respectively ~ 1.6 × 10^5^ µm^2^ and ~ 5 × 10^5^ µm^2^ after 6 days of culture. It has to be stated that this analysis shows the evolution of spheroid sizes but not the actual number of cells that compose the spheroids. In addition, the count of HGT-1 cells, recovered from monocellular or bicellular spheroids, showed no significant differences at the same age of spheroids (Supplementary Fig. 8). In this mixed model, cells were firmly attached to each other and were hard to dissociate by mechanical force. These data indicated that the presence of fibroblasts, in 1:1 ratio with cancer cells, promoted assembly of highly compact 3D cell structures (Supplementary Videos, bicellular spheroids formation until day 8). In addition, AGS + CAF spheroids, assembled from 500 AGS + 500 CAF were smaller and more compact than monocellular 500 AGS spheroids, similarly to HGT-1 + CAF bicellular spheroids (data not shown).

**Figure 7:**
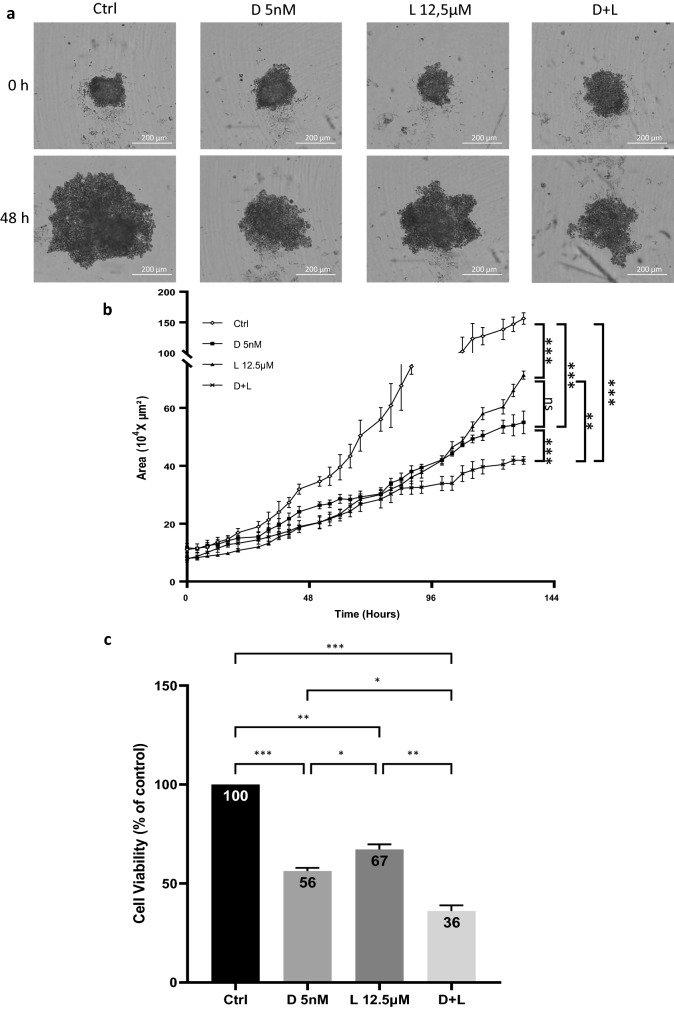
Effects of drug treatments on bicellular MCTS. (**a**) Bright field photographs of 6 days-old HGT-1 + CAF spheroids at 0 h and 48 h of treatment with 5 nM docetaxel (D 5 nM), 12.5 µM lovastatin (L 12.5 µM) and 5 nM docetaxel + 12.5 µM lovastatin (D + L), captured with the Incucyte live imaging. (**b**) Real-time growth monitoring (Area 10^4^ × µm^2^) of mixed spheroids formed from 250 HGT-1 + 250 CAF was performed using the Incucyte live imaging and analysis system. Single treatments with 5 nM docetaxel (D 5 nM) (black squares), 12.5 µM lovastatin (L 12.5 µM) (black triangles) and combined treatment with 5 nM docetaxel + 12.5 µM lovastatin (D + L) (black X) were applied on 6 days-old MCTS and compared to control (white lozenges). The results are shown as the mean ± SD of n = 3 independent experiments with four technical replicates in each. ns, *p* > 0.05; **p ≤ 0.01; ***p ≤ 0.001, one-way ANOVA followed by Tukey analysis.(**c**) The variation of bicellular MCTS viability was determined by the MTT assay after 48 h of treatment by 5 nM docetaxel (D 5 nM), 12.5 µM lovastatin (L 12.5 µM) and combined treatment with 5 nM docetaxel + 12.5 µM lovastatin (D + L). The results are shown as the mean ± SD of n = 3 independent experiments with four technical replicates in each. *p ≤ 0.05; **p ≤ 0.01; ***p ≤ 0.001, one-way ANOVA followed by Tukey analysis.

Then, we treated 6 days-old HGT-1 + CAF bicellular MCTS by D 5 nM, L 12.5 µM and D + L. Reduction of spheroids size was shown upon treatments. As shown by bright field photographs of spheroids in Fig. 7a, the association of docetaxel and lovastatin triggered a stronger size reduction of MCTS compared to single docetaxel treatment after 48 h. This was also reflected over time up to 144 h (Fig. 7b). To determine if this size reduction of MTCS was link to cell death, we performed MTT assays from 6 days-old bicellular spheroids after 48 h of treatment. D 5 nM showed a stronger cell killing activity (viability reduced by 44%, *p* < 0.001) than L 12.5 µM (viability reduced by 33%, *p* < 0.01). The association of D + L had the largest effect on cell viability reduction (up to 64%, *p* < 0.001) (Fig. 7c). We have also examined cell death inducing effects of D 5 nM, L 12.5 µM and D + L on 6 days-old AGS + CAF bicellular spheroids using an MTT assay (Supplementary Fig. 9). Combined treatment had a cumulative effect on cell viability, similarly to HGT-1 MCTS.

## Discussion

Although treatment efficacy has improved during the past decade, survival rates of GC patients remain low^47^. The limitations of 2D cell culture model that lacks tumor complexity and direct pathophysiology relevance may be one of the main causes of the poor rate of cancer drugs entering early clinical trials, and even more so, become marketed drugs^48^. Novel preclinical models such as 3D cultures may be more predictive than 2D cultures for cancer therapy research^49^. Several distinct factors between 2D and 3D cultures, such as metabolic state and expression of drug resistance transporters could affect the activity of anti-cancer drugs^29^.

Following on our previous studies showing that blunting the mevalonate pathway with lovastatin amplified the apoptotic response brought about by docetaxel^42^, we asked whether such results might also apply to cells grown in 3D as spheroids. This was motivated by the fact that cell–cell interactions might be different between 2D and 3D culture conditions. Although this may sometimes be the case^44,50^, the present results showed that in both culture conditions, the lovastatin + docetaxel combination proved to be similarly able to trigger efficient apoptosis, to a larger extent than single drug treatments.

Here, HGT-1 and AGS cells were found to spontaneously form spheroids in ULA round-bottom microplates with high reproducibility. These spheroids were used for drug toxicity evaluation. We made use of high-throughput image microscopy (Incucyte) to analyze several growth-impairing conditions of spheroids, as reported^51^. Protein and gene expression profiles of MCTS are reportedly more similar to those of tumors than 2D cancer cells^52,53^. In addition, 3D cell models are usually more resistant to cytotoxic treatments compared to cells in 2D cultures^54,55^. This may have applied also here, as cells derived from 6 days-old spheroids were slightly less sensitive to the drugs than 2D-cultured cells after 48 h of treatment (Supplementary Fig. 10). However, it is important to consider spheroid sizes when comparing therapeutic responses^56^. The spheroids generated here were homogenous and reached sizes in this range after 6 days. Although AGS spheroids were more compact than HGT-1 spheroids, both showed comparable sensitivities to the drugs or their combinations. Following drug treatments, we obtained quite overlapping results, by both the MTT assay and the live microscopy analysis system (Figs. 3, 4).

Cancer cells are strongly influenced by their microenvironment, which modulates local tumor progression and has a significant impact on therapy^57^. The co-culture of tumor cells with CAF in MCTS permits to reproduce, at least in part, the interactions between different cell types, which are known to affect disease progression and the efficacy of anticancer therapies^58,59^. Several studies showed that distinct signaling pathways may be activated in 2D versus 3D culture, in the presence or absence of stromal cells upon treatment with RAF inhibitors^60^, anti-androgens^61^, cetuximab, trastuzumab, vorinostat or everolimus^62^ and doxorubicin^63^. Here, we developed an in vitro GC 3D direct co-culture model using human GC cells and GC-associated CAF. From day 2 of co-culture, spheroids assumed a tight shape, as compared to pure HGT-1 spheroids. This had no detrimental impact on drug response, as the combination of docetaxel and lovastatin showed strong cytotoxicity in this setup also (Fig. 7).

Overall, the cytotoxic responses observed here in 2D or 3D models were presumably mostly due to some of the many changes induced by lovastatin at the transcriptomic and metabolomics levels, rather than to docetaxel that had very limited effects^42,64^. Because the two drugs share no common mechanism of action, we believe, that they acted independently to trigger cell death, rather than activating the same death pathway.

Our results showed, by 3 analytical methods, from 2D cell cultures to 3D, that the combination of a *bona fide* anticancer agent, like docetaxel, and lovastatin deserved much interest. Real-time monitoring of drug response of 3D tumor spheroids proved to be a quite sensitive and reliable approach to determine the overall cell toxicity, both in mono-cellular and in bi-cellular spheroids. This toxicity resulted in apoptosis, as shown by enhanced nuclear fragmentation (revealed by Hoechst 33342 nuclear staining) and increased Caspases 3/7 activities. As an extension to this study, it could be envisioned to look at the response of GC organoids, i.e., tumor fragments recovered right after surgery of human patients, to in vitro treatments with lovastatin and/or docetaxel. This might allow an even better application of 3D model screening assays to improve further the efficiency of drug screening. Transposed to a clinical setup, these results may lead to propose the association of lovastatin with docetaxel for the treatment of patients with GC. One evident advantage of this combination therapy would be to use lower drug doses than usually implemented, thereby reducing potential toxic side-effects, like those that have been reported for patients regularly taking statins or for cancer patients undergoing taxane anticancer therapy.

## Methods

### Cell culture

HGT-1 human gastric cancer cells (a gift of Pr. C. Laboisse, Nantes University hospital, France) were grown at 37 °C under a humidified atmosphere with 5% CO_2_ in Dulbecco's modified Eagle's medium (DMEM) (Corning, MA, USA), containing 4.5 g/L glucose and supplemented with 5% fetal bovine serum (FBS) (Gibco-Invitrogen, Cergy-Pontoise, France) without antibiotics (complete medium). AGS human gastric cancer cells (from the American Tissue Type Collection, ref. ATCC® CRL-1739™) were grown in the same medium supplemented with 10% FBS.

### Lentiviral infection

The self-inactivating HIV-1-based lentiviral vector, pRRL-sin-MND-Tomato-IRES-Puro was purchased from VectUB (vectorology platform, University of Bordeaux, France). The vector expresses RedTomato and co-expresses the puromycin resistance gene. 40,000 HGT-1 cells were infected with lentiviruses (at a multiplicity of Infection of 2) and selected with puromycin (1 µg/mL). We obtained RedTomato fluorescent HGT-1 populations.

### Preparation of mono- and bi-cellular tumor cells spheroids

Two-Dimension cultured HGT-1 or AGS cells were collected and used to generate spheroids by seeding 500 or 1000 cells/well (in 200 µL of complete medium), respectively, in ultra-low attachment 96-well round bottom microplates (Corning, Amsterdam, Netherlands). Following cell aggregation, ~ 550 µm diameter spheroids were obtained after 6 days of incubation under standard culture conditions.

To obtain bi-cellular spheroids, cells of each type were added together in 1:1 ratio (250 HGT-1 + 250 CAF or 500 AGS + 500 CAF).

### Isolation, characterization and maintenance of fibroblast cultures

The experimental protocols were approved by the Ethics Committee of Brest University Hospital (headed by Pr J.M. Boles). Informed consent was obtained from all the patients. Samples were processed in the Anatomy and Pathology department of Brest University Hospital. From these, we isolated CAF following growth in primary tissue culture. We confirm that all methods were carried out in accordance with relevant guidelines and regulations and that all experimental protocols were approved by Brest University Hospital in respect with French regulations.

Cancer Associated Fibroblasts were obtained from a GC patient-ablated tumor after tissue dissociation with collagenase and growth in culture in DMEM (Corning, MA, USA) containing 4.5 g/L glucose supplemented with 10% FBS (Gibco-Invitrogen, Cergy-Pontoise, France). After about 10 days in culture, no more epithelial cells adhered to the dish and fibroblasts cells emerged and kept growing. Cells were then passaged every 2 weeks. They were used for RNA preparation to analyze their expression of fibroblast-specific transcripts at early (< 7) passages and 70–90% confluence, so as to maintain their in vivo characteristics^65^.

### Drugs

Lovastatin and Docetaxel were from TCI Europe (Belgium) and Sanofi Aventis (France), respectively. Appropriate ranges of concentrations were chosen from previous dose–response studies in 2D^42^. Lovastatin was dissolved and diluted in dimethyl-sulfoxide (DMSO). The final concentration of DMSO used in culture did not exceed 0.4%, a concentration that had no overt cytotoxic effect *per se*. Docetaxel was used after dilution in 0.9% sodium chloride.

### MTT assay

To evaluate 2D-cell viability, cells were seeded into 96-well culture plates at a density of 5000 cells/well and grown in 100 µL of medium. After 24 h, the medium was replaced by 100 µL of fresh medium containing the drugs. After 36 or 48 h of treatment, 10 µL of MTT labeling reagent (Millipore) dissolved in Phosphate Buffered Saline were added to each well (MTT reagent final concentration was 0.5 mg/ml). Plates were incubated for 2 h at 37 °C. Then, formazan crystals were dissolved by adding 100 µL of solubilization solution (Isopropanol, Triton X-100 10%, 0.1 M HCl) into each well. MTT reduction was quantified by measuring the light absorbance at 570 nm using an absorbance microplate reader (Multiskan Spectrum microplate spectrophotometer, ThermoFisher).

MTT assay for the MCTS cultures was carried out with slight modifications of the standard protocol. After 48 h of treatment, 100 µL (1/2 of total) of medium were carefully removed. The spheroids were then dissociated mechanically before the addition of 10 µL MTT reagent into each well. The plates were incubated for 2 h at 37 °C. After incubation, formazan crystals were dissolved by adding 100 µL of solubilization solution into each well. Then 150 µL of medium from each well containing the MCTS culture were transferred to a new, flat-bottom 96-well plate. Absorbance was recorded as described for 2D cells.

### Multicellular tumor spheroids size measurement

An Incucyte S1 live‐cell analysis system (Sartorius, Essen Bioscience), placed inside a conventional tissue culture incubator at 37 °C with 5% CO_2_, was used for real-time imaging of spheroids. Images of each spheroid were taken using a 4× phase contrast lens, every 4 h for 11 days, and each condition was run in quadruplicate. Each well contained a single spheroid settled at the center. Images were analyzed and data were generated using the spheroid automated software algorithm functions from Incucyte 2019B Rev2 software (Sartorius, Essen Bioscience) where virtual masks were created to surround spheroids. The size of spheroids was calculated as the largest object area in each image.

### Evaluation of the RedTomato fluorescence intensity in spheroids

The fluorescence signal generated from HGT-1 RedTomato-labeled cells, cultured as spheroids, was analyzed in the Incucyte system. MCTS were imaged every 4 h with 1 image/well in phase contrast and Red fluorescence channels (400 ms exposure) using a 4× lens, each condition being run in quadruplicate. Automated real‐time assessment by live‐cell analysis was measured as red area generated by viable HGT-1 RedTomato-labeled cells. The fluorescence is proportional to the number of intact viable cells in the well for all RedTomato cells with normalization on contrast phase areas. Data were analyzed using the spheroid software functions from Incucyte 2019B Rev2 software.

### Incucyte Annexin V apoptosis assay

The Incucyte S1 live‐cell analysis system was used to determine apoptosis levels of 3D cultured HGT-1 cells treated with the drugs. HGT-1 spheroids were cultured for 6 days and annexin V Red reagent (Sartorius, Essen Bioscience) was added at the same time as drugs. Throughout the assay, both phase and fluorescent images were collected using phase contrast and Red fluorescence channels (400 ms exposure) with a 4× lens. One image was taken every 4 h for 5 days, and each condition was run in quadruplicate. Automated real‐time assessment by live‐cell analysis was measured as red area for all cells stained red with Annexin V Reagent normalized to contrast phase area. Images were analyzed and data were generated using the spheroid automated software algorithm functions from Incucyte 2019B Rev2 software.

### Statistics

Statistical analysis was performed using GraphPad Prism. Data are presented as the mean ± S.D and experiments were repeated at least three times. *p* Values were calculated using one-way ANOVA with Tukey analysis. The results were considered significant for *p < 0.05, **p < 0.01 and ***p < 0.001.

## Supplementary Information

Supplementary Information 1.

Supplementary Information 2.

Supplementary Information 3.

Supplementary Information 4.
