# Supplementary material for: Gastric cancer cell death analyzed by live cell imaging of spheroids
Source: Sci Rep. 2022 Jan 27;12:1488. doi: 10.1038/s41598-022-05426-1 (PMC8795446; doi:10.1038/s41598-022-05426-1)

**Supplementary Figure 1.**

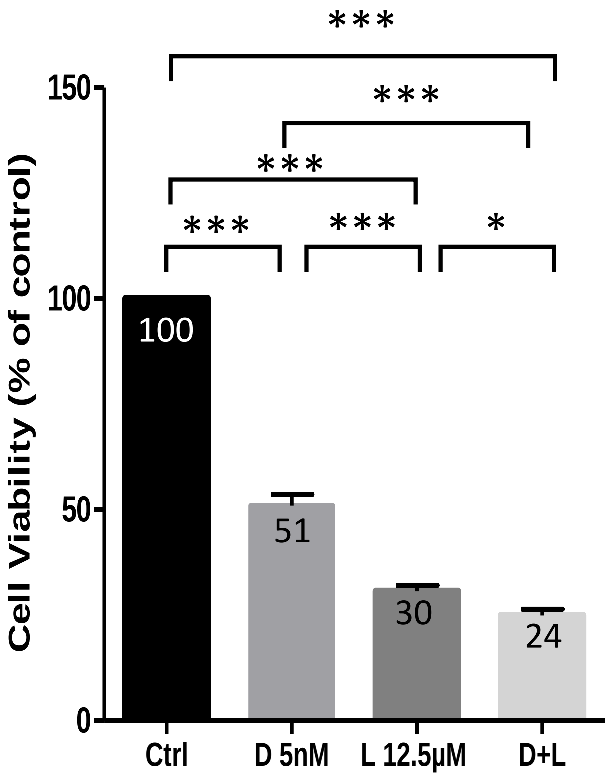

**Supplementary Figure 2.**

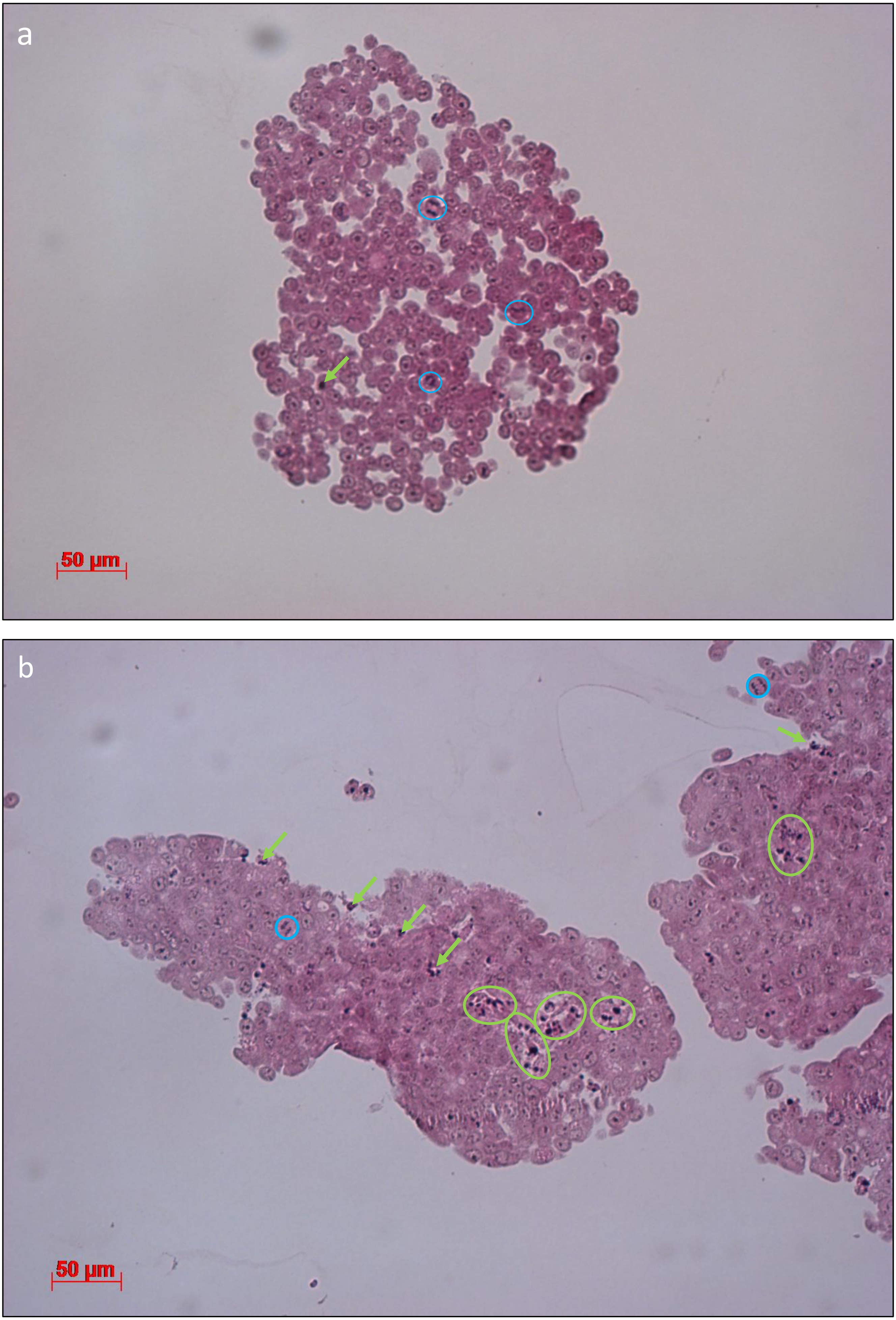

**Supplementary Figure 3.**

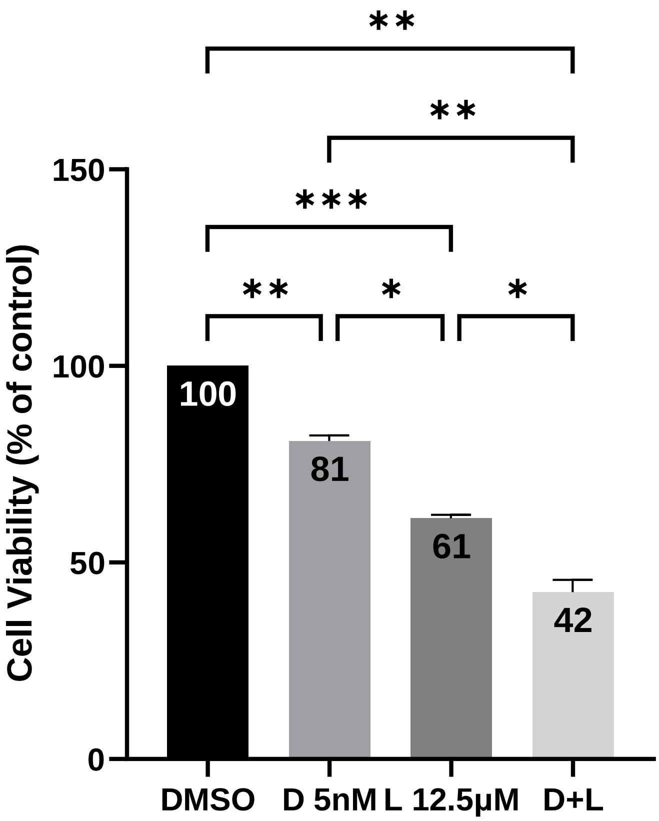

**Supplementary  Figure 4.**

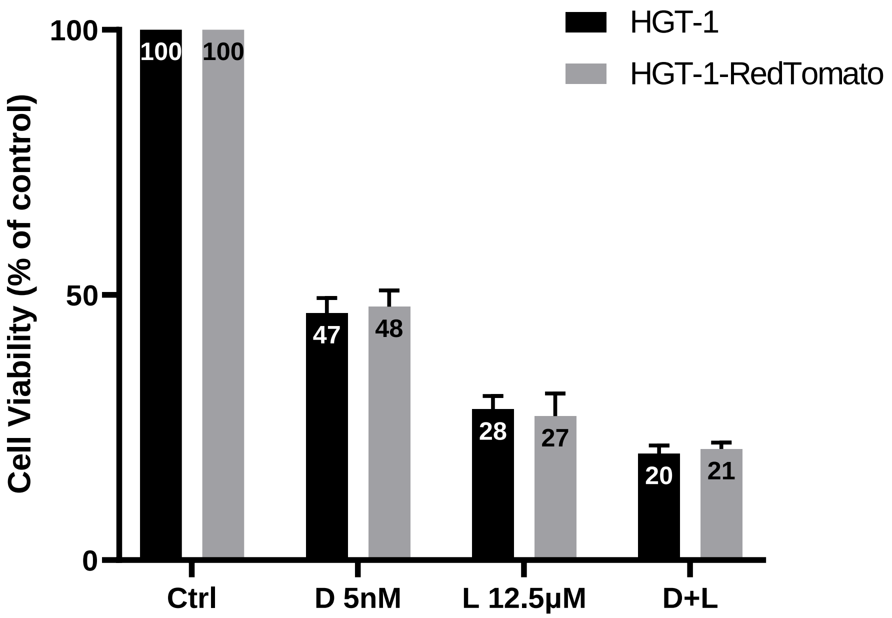

**Supplementary Figure 5.**

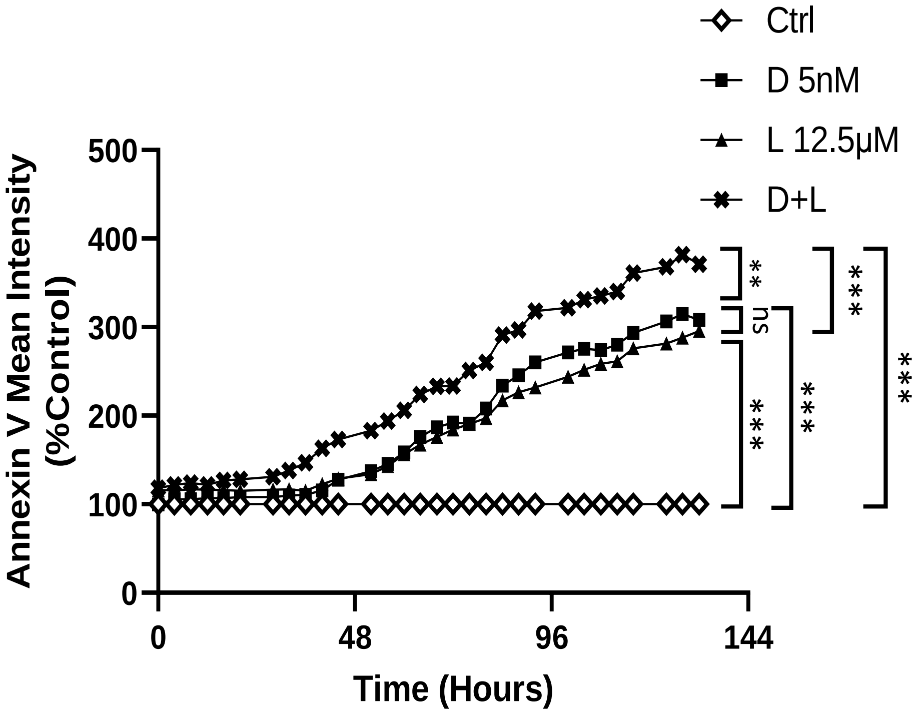

## Supplementary Figure 6.

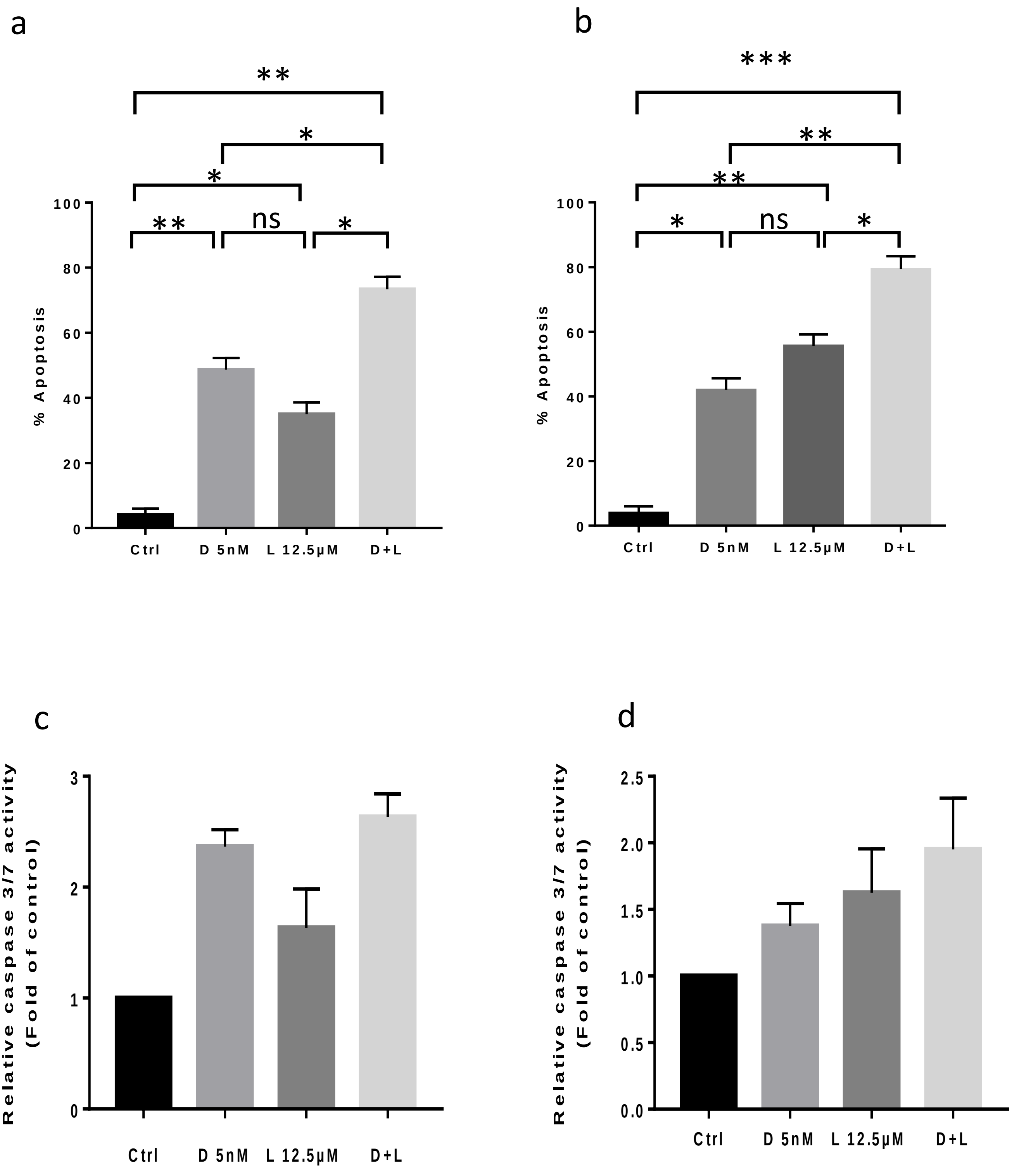

**Supplementary Figure 7.**

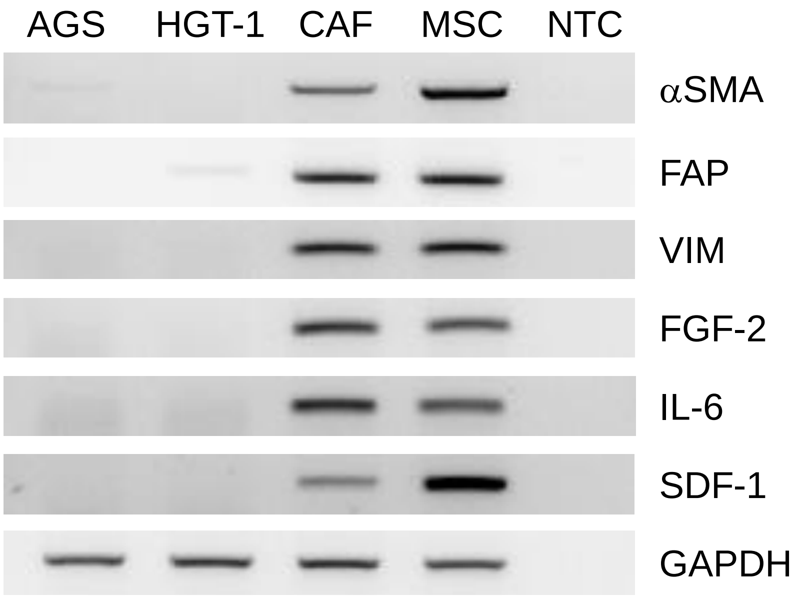


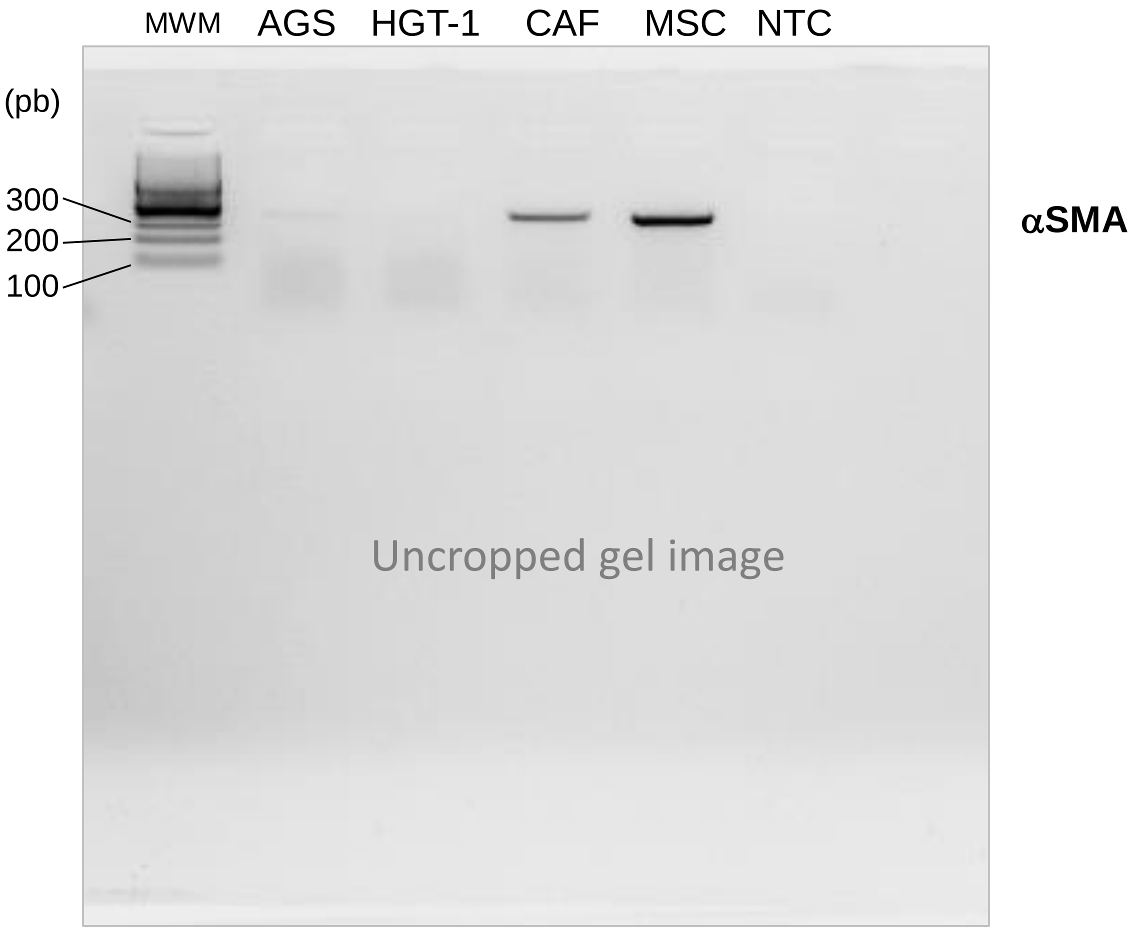

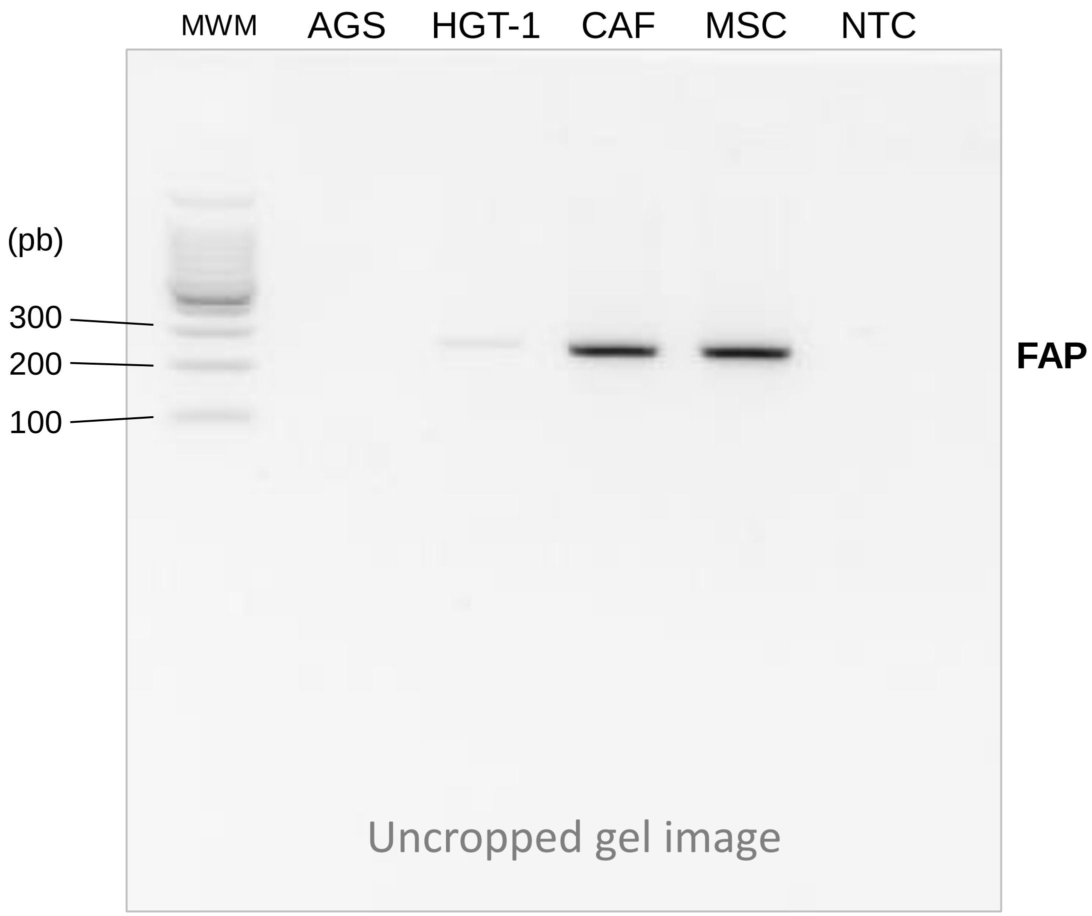
MWM
AGS
HGT-1
CAF
MSC
NTC
(pb)
300
200
100
FAP
Uncropped gel image

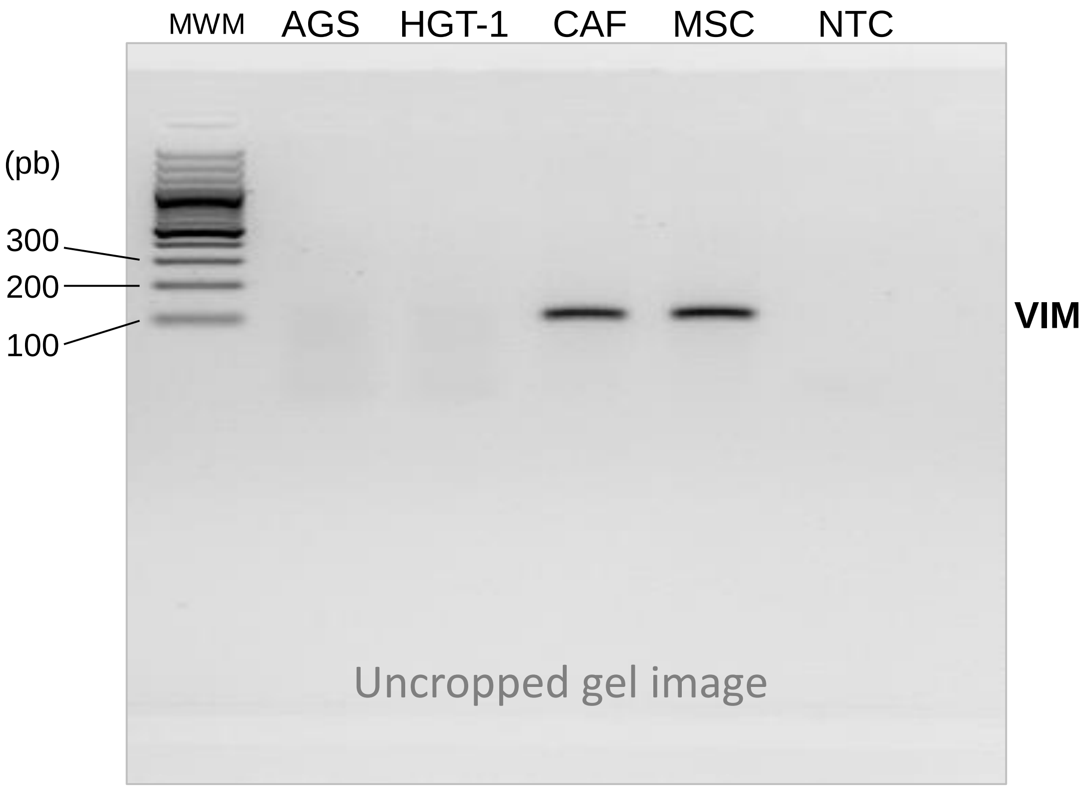
MWM
AGS
HGT-1
CAF
MSC
NTC
(pb)
300
200
100
VIM
Uncropped gel image

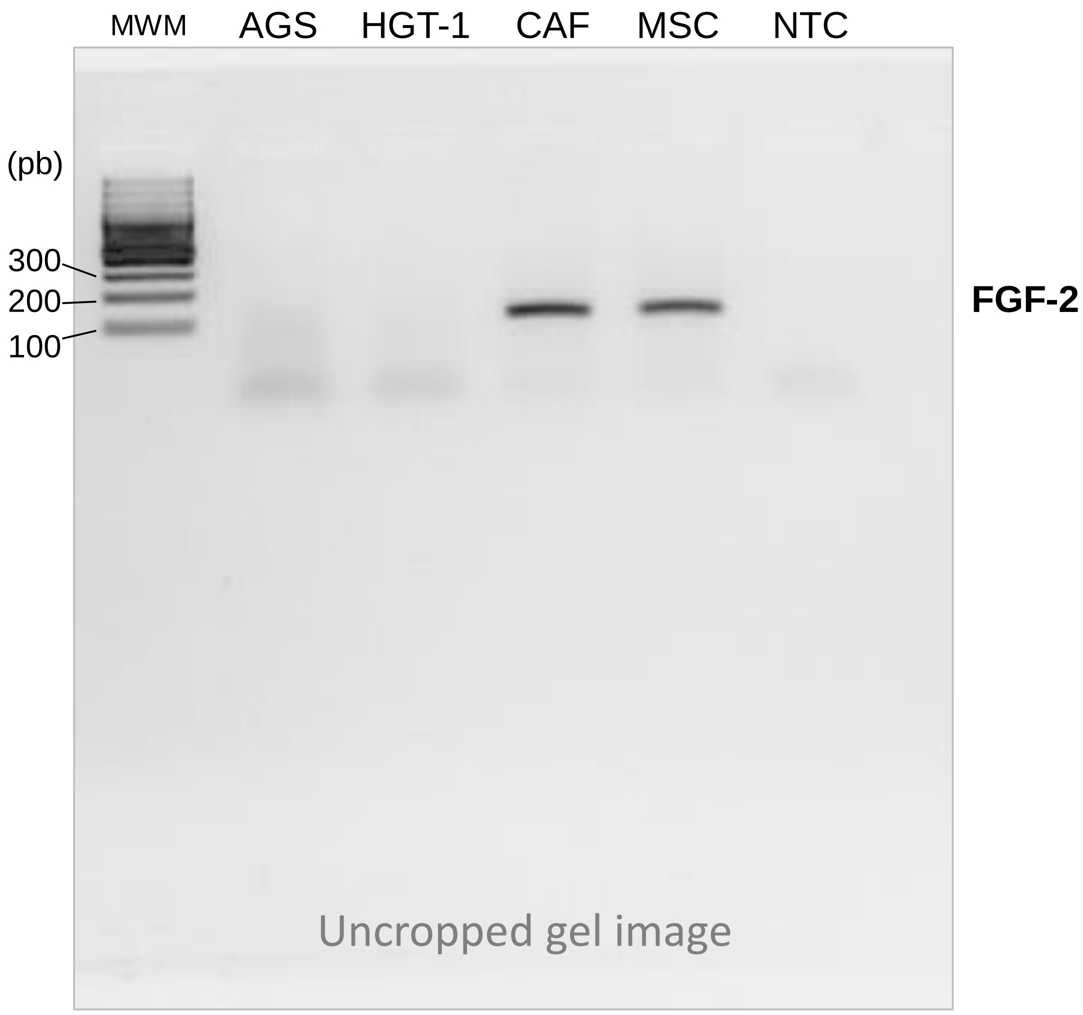
MWM
AGS
HGT-1
CAF
MSC
NTC
(pb)
300
200
100
FGF-2
Uncropped gel image

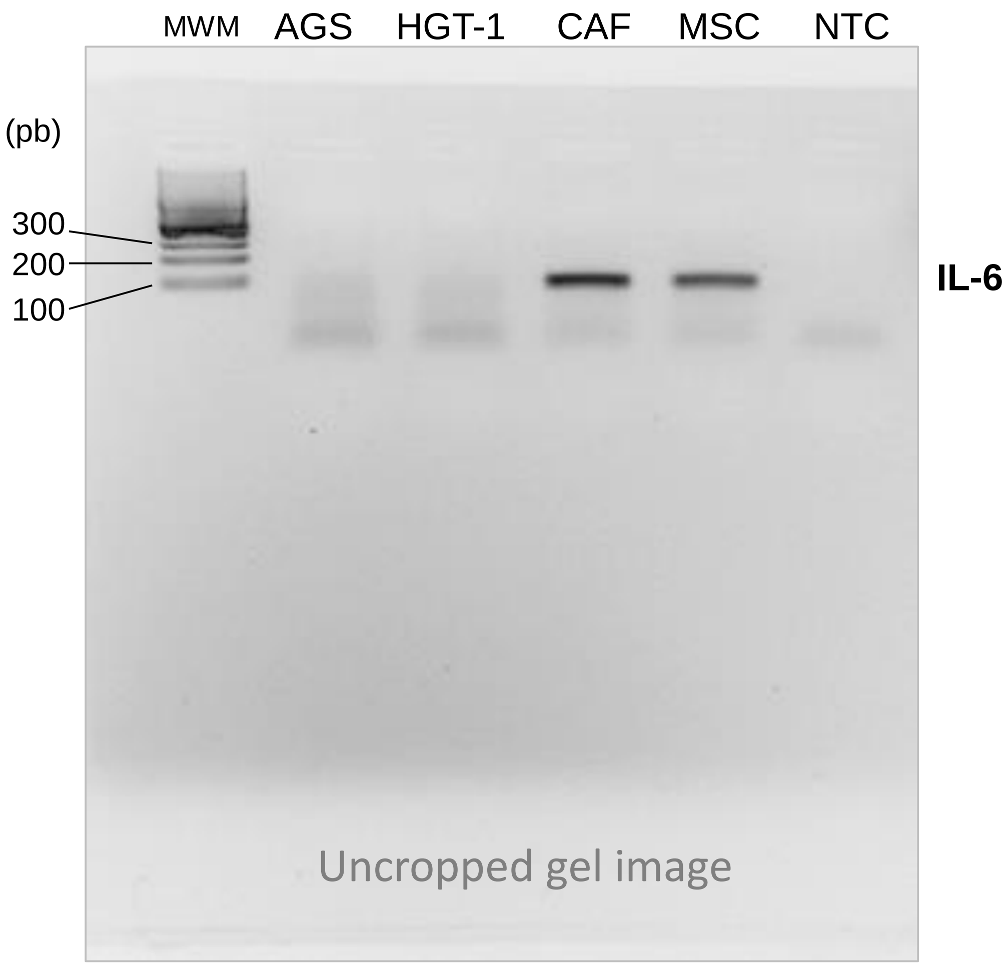
MWM
AGS
HGT-1
CAF
MSC
NTC
(pb)
300
200
100
IL-6
Uncropped gel image

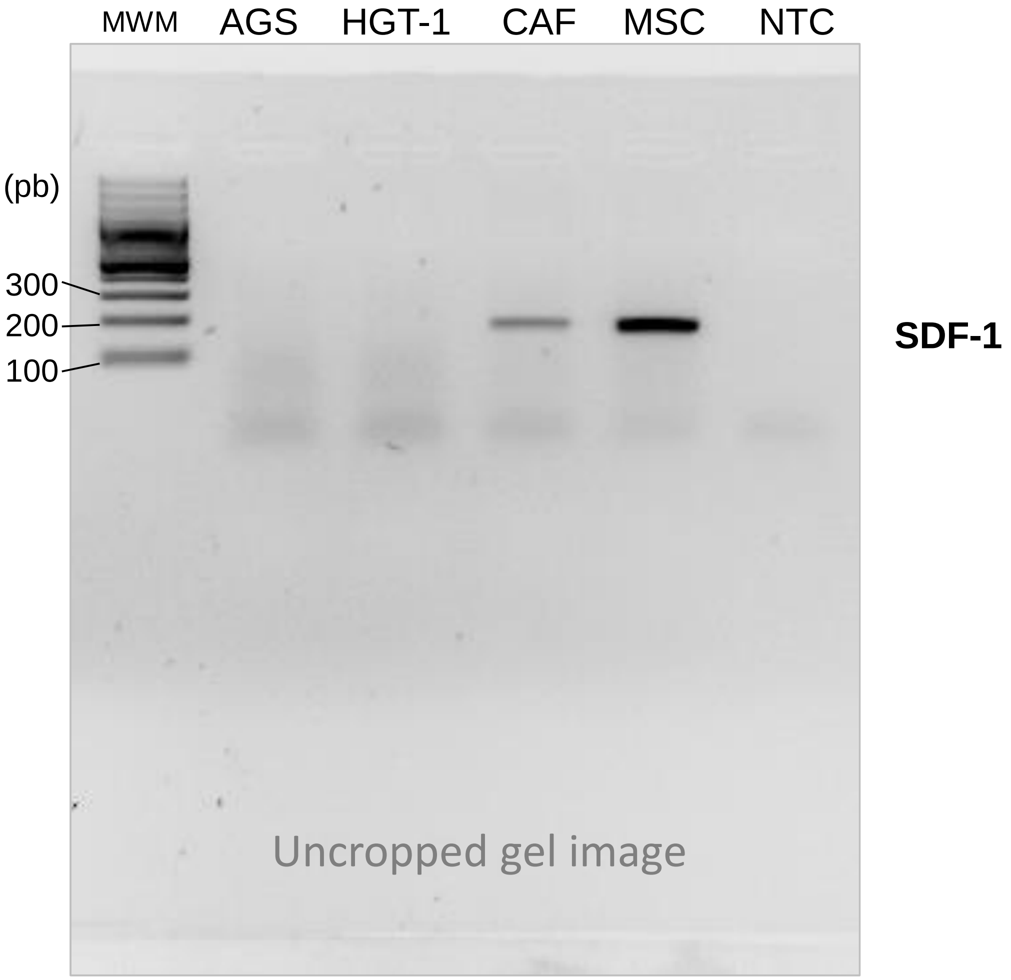
MWM
AGS
HGT-1
CAF
MSC
NTC
(pb)
300
200
100
SDF-1
Uncropped gel image

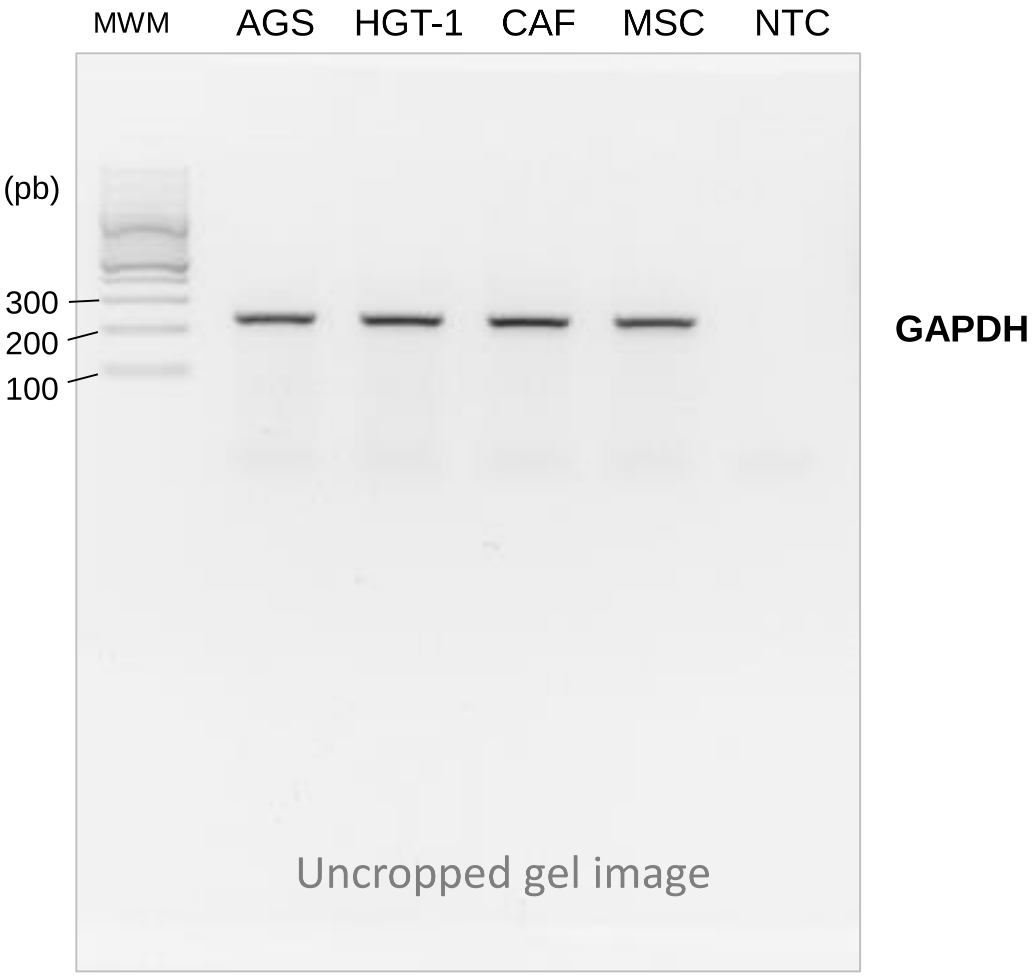
MWM
AGS
HGT-1
CAF
MSC
NTC
(pb)
300
200
100
GAPDH
Uncropped gel image

## Supplementary Figure 8.

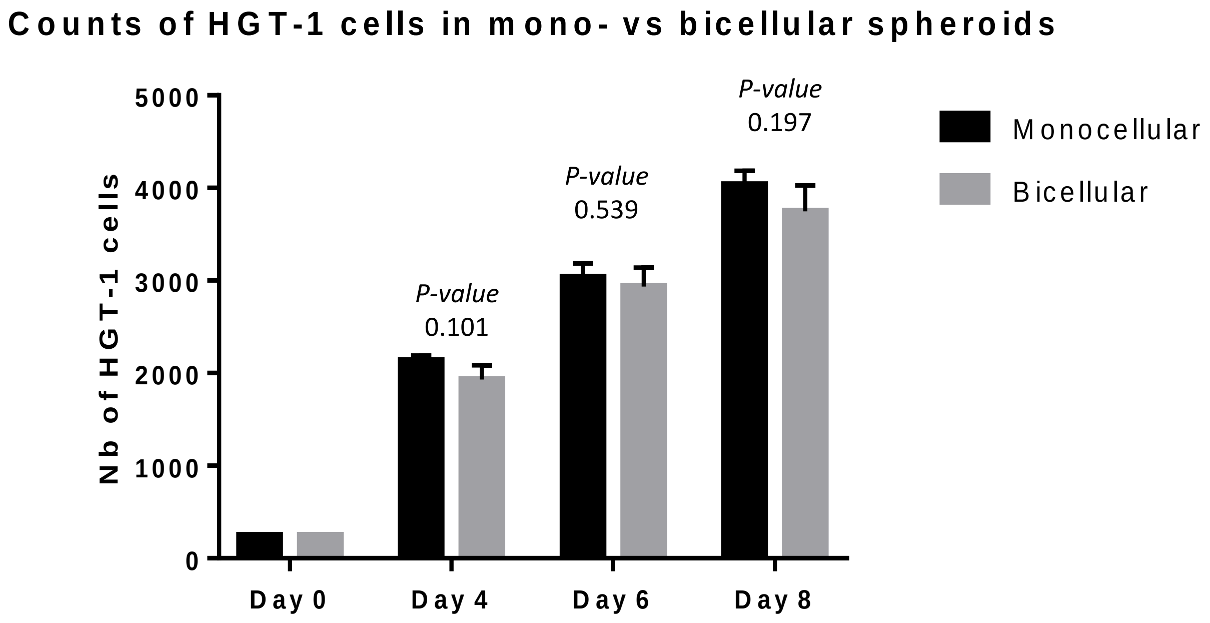

# Supplementary Figure 9.

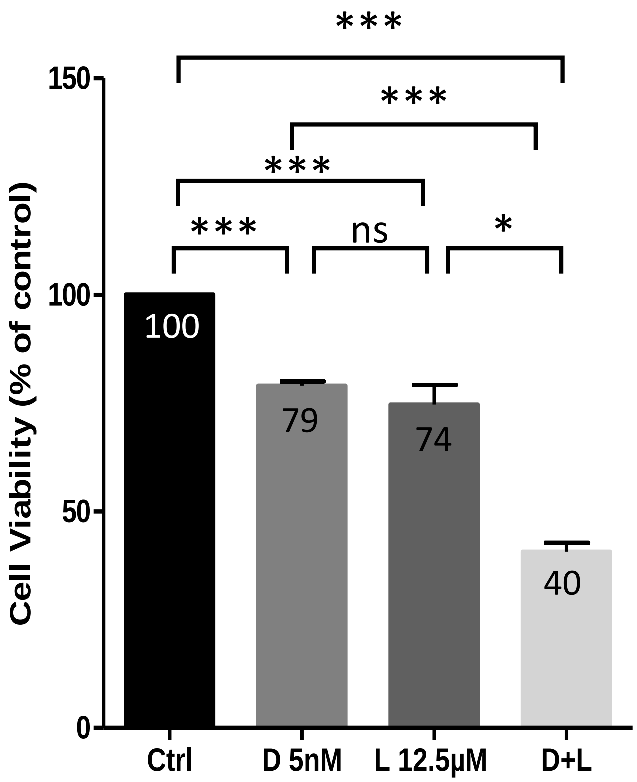

**Supplementary Figure 10.**

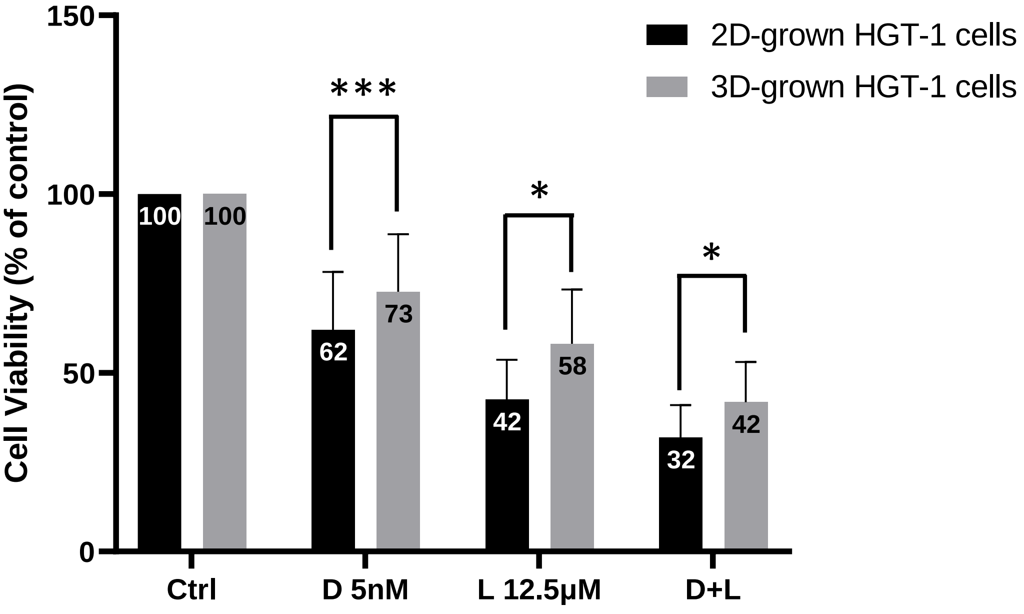

Supplement: Supplementary file 3 — Supplementary Information 3. [file 41598_2022_5426_MOESM3_ESM.pdf]
