# Supplementary material for: Gastric cancer cell death analyzed by live cell imaging of spheroids
Source: Sci Rep. 2022 Jan 27;12:1488. doi: 10.1038/s41598-022-05426-1 (PMC8795446; doi:10.1038/s41598-022-05426-1)
Supplement: Supplementary file 4 — Supplementary Information 4. [file 41598_2022_5426_MOESM4_ESM.docx]

Gastric cancer cell death analyzed by live cell imaging of spheroids

George Alzeeb, Danielle Arzur, Valérie Trichet, Matthieu Talagas, Laurent Corcos, Catherine Le Jossic-Corcos

**Supplementary Materials and Methods**

**Determination of apoptotic nuclear fragmentation and caspase activation**

After 48 h of treatment, the spheroids were dissociated mechanically before they were stained with Hoechst 33342 (Sigma) at 10 µg/ml in complete medium for 10 min at 37°C. Nuclei of apoptotic cells with fragmented chromatin were brightly stained. At least 400 cells were counted for each sample, and the percentage of total apoptotic cells was calculated.

The activity of caspase-3 and caspase-7 was determined with the luminescent Caspase-Glo 3/7 Assay kit (Promega, France) according to the manufacturer’s instructions. Briefly, after treatment of 6 days-old spheroids, an equal volume of Caspase-Glo 3/7 reagent was added to the sample in the assay well. Samples were incubated at room temperature for 1 hour and the enzyme activity was measured with a luminometer (Fluoroskan ascent FL, Thermo electron corporation).

**Histological analysis of HGT-1 and AGS spheroids**

Spheroids were fixed with buffered formalin for 1 hour at room temperature, embedded in a gel matrix (7401151, Cytoblock Replacement Reagents, Thermo Scientific, Waltham, USA) according to the manufacturer’s instructions, prior to dehydration and paraffin-embedding. Five µm thick sections were stained with haematoxylin, eosin and saffron. Images acquisition was performed with AxioVision 4.8 software (Zeiss, Oberkochen, Germany).

**Drug resistance assays**

Sensitivity to drugs was assessed using the MTT assay. Sixty 6 days-old spheroids were dissociated mechanically and 5000 healthy MCTS-dissociated cells or parental cells per well were plated in 96-well plates in 100 µL of DMEM supplemented with 5% FBS with or without drugs (docetaxel, lovastatin). To analyze drug sensitivity of MCTS-dissociated cells, as compared to 2D-only grown cells, we treated both cell populations with 5nM docetaxel +/- 12.5µM lovastatin. Forty-eight hours after treatment, 10 µL of the MTT labeling reagent dissolved in Phosphate Buffered Saline were added to each well (final concentration 0.5 mg/ml). The plates were incubated for 2h at 37 °C. After incubation, formazan crystals were dissolved by adding 100 µL of the solubilization solution (Isopropanol, Triton X-100 10%, 0.1M HCl) into each well. MTT reduction was quantified by measuring the light absorbance at 570 nm using an absorbance microplate reader (Multiskan Spectrum microplate spectrophotometer, ThermoFisher). Each test was repeated three times in quadruplicate determinations.

**RNA extraction and RT-PCR analysis**

Total RNA was isolated using TRIzol (Invitrogen) according to the manufacturer’s protocol. Samples of 2 µg of total RNA were reverse-transcribed using random hexamers and MMLV (Moloney-murine-leukaemia virus) reverse transcriptase (New England Biolabs). RT reactions (1 µL out of a 25 µL reaction volume) were used as templates for PCR experiments.

The characteristics of primers and amplicons size are in the table below:


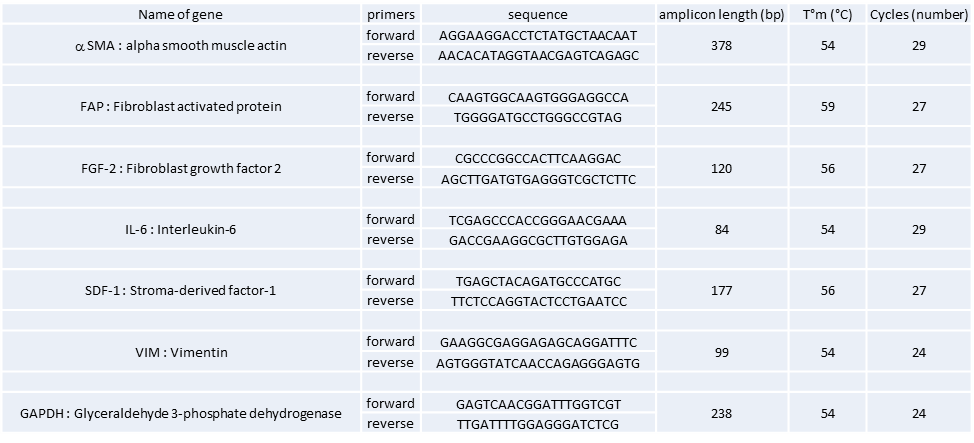


**Supplementary Figures legends**

**Supplementary Figure 1. Toxicity of docetaxel and lovastatin in human gastric cancer AGS cells in 2D culture.**

The cells were incubated at 37 °C for 48 h upon treatment with 5nM docetaxel (D 5nM), 12.5µM lovastatin (L 12.5µM) or 5nM docetaxel + 12.5µM lovastatin (D+L). Cell viability was measured by the MTT assay. The results are shown as the mean ± SD of n=3 independent replicates with four technical replicates in each. **p* ≤ 0.05; ****p* ≤ 0.001, one-way ANOVA followed by Tukey analysis.

**Supplementary Figure 2. Histological sections of HGT-1 and AGS spheroids.**

HGT-1 cells (a) are grouped into loosely cohesive clusters. Mitotic figures are visible (blue circles). A few isolated necrotic cells are present (green arrow).

AGS cells (b) are grouped into cohesive clusters. Necrotic areas are present in the center of the clusters (green arrows and circles). A few mitotic figures are visible (blue circle).

**Supplementary Figure 3. Cytotoxicity of docetaxel and lovastatin for AGS spheroids**

Cell viability was determined by the MTT assay after 48h of treatment with 5nM docetaxel (D 5nM), 12.5µM lovastatin (L 12.5µM) or 5nM docetaxel + 12.5µM lovastatin (D+L) of six days-old AGS spheroids. The results are shown as the mean ± SD of n=3 independent replicates with four technical replicates in each. **p* ≤ 0.05; ***p* ≤ 0.01; ****p* ≤ 0.001, one-way ANOVA followed by Tukey analysis.

**Supplementary Figure 4. Cell toxicity of drugs on RedTomato-labelled HGT-1 cells.**

The cells were incubated at 37 °C for 48h upon treatment by 5nM docetaxel (D 5nM), 12.5µM lovastatin (L 12.5µM) or 5nM docetaxel + 12.5µM lovastatin (D+L). Cell viability was measured by the MTT assay. The results are shown as the mean ± SD of n=3 independent replicates with four technical replicates in each.

**Supplementary Figure 5. Real-time apoptosis induction by docetaxel and lovastatin in HGT-1 spheroids.**

Real-time Annexin V fluorescent reagent (Incucyte) measurements are shown. Six days-old MCTS were treated with 5nM docetaxel (D 5nM) (black squares), 12.5µM lovastatin (L 12.5µM) (black triangles) and the combined treatment 5nM docetaxel + 12.5µM lovastatin (D+L) (black X). The control condition is shown as white lozenges. The Annexin V reagent was added at the same time as the drugs. The results are shown as the mean of n=3 independent replicates with four technical replicates in each. ns *p* > 0.05; ***p* ≤ 0.01; ****p* ≤ 0.001, one-way ANOVA followed by Tukey analysis.

**Supplementary Figure 6. Apoptosis induction by docetaxel and lovastatin in HGT-1 and AGS spheroids**

After 48 h of treatment by 5nM docetaxel (D 5nM), 12.5µM lovastatin (L 12.5µM) or 5nM docetaxel + 12.5µM lovastatin (D+L), apoptosis of 6 days-old HGT-1 (a) or AGS (b) spheroids was evaluated by Hoechst 33342 staining of fragmented nuclei. The results are shown as the mean ± SD of n=3 independent replicates with four technical replicates in each. ns *p* > 0.05; **p* ≤ 0.05; ***p* ≤ 0.01; ****p* ≤ 0.001, one-way ANOVA followed by Tukey analysis.

Caspase 3/7 activity (caspase-Glo^TM^ 3/7 assay, Promega) was determined after 24h of treatment by 5nM docetaxel (D 5nM), 12.5µM lovastatin (L 12.5µM) or 5nM docetaxel + 12.5µM lovastatin (D+L), in 6 days-old HGT-1 (c) or AGS (d) spheroids. The results are mean ± SD of three wells; they should be considered as indicative since they are from a single experiment.

**Supplementary Figure 7. Gene expression levels in HGT-1, AGS, CAF and MSC**

RNA levels of fibroblast markers were analyzed by RT-PCR (see materials and methods) in HGT-1 cells, AGS cells, in CAF and in mesenchymal stem cells (MSC). NTC: no template control, negative control for PCR. Alpha-smooth muscle actin (α-SMA), fibroblast activation protein (FAP), vimentin (VIM), fibroblast growth factor-2 (FGF-2), interleukin-6 (IL-6), stromal-derived factor-1 (SDF-1), glyceraldehyde-3-phosphate dehydrogenase (GAPDH). MWM: 100pb DNA ladder molecular weight marker (Promega).

**Supplementary Figure 8. Counts of HGT-1 cells in mono- vs bicellular spheroids**

GFP-labelled HGT-1 cells were counted following dissociation of monocellular or bicellular spheroids up to 8 days. The results are shown as the mean ± SD of n=3 independent replicates. *P-value* were issued from Student’s t-test.

**Supplementary Figure 9. Effects of drug treatments on bicellular MCTS (AGS+CAF)**

The variation of mixed spheroids (500 AGS + 500 CAF) viability was determined by the MTT assay after 48 h of treatment by 5nM docetaxel (D 5nM), 12.5µM lovastatin (L 12.5µM) and combined treatment with 5nM docetaxel + 12.5µM lovastatin (D+L). The results are shown as the mean ± SD of n=3 independent replicates with four technical replicates in each. ns *p* > 0.05; **p* ≤ 0.05; ****p* ≤ 0.001, one-way ANOVA followed by Tukey analysis.

**Supplementary Figure 10. Effects of drugs in HGT-1 2D cells compared to HGT-1 cells recovered from MCTS and re-grown in 2D**

MTT assay was used to analyze drug toxicity for 48h in HGT-1 2D cells and in HGT-1 cells recovered from six days-old HGT-1 MCTS. The results are shown as the mean ± SD of n=3 independent replicates with four technical replicates in each. **p* ≤ 0.05; ****p* ≤ 0.001, Student’s t-test.

**Supplementary Movies**

**Effects of docetaxel and lovastatin treatments on the growth of HGT-1 spheroids**. Movies show real-time HGT-1 spheroid growth after treatment with 5nM docetaxel, 12.5µM lovastatin and 5nM docetaxel + 12.5µM lovastatin (D+L), captured with the Incucyte live imaging microscope. Day 0 correspond to 5 days-old spheroids. Drugs where added at day 1: on 6 days-old spheroids.

**Bicellular HGT-1 + CAF spheroids formation.** Movies show real-time 250 HGT-1, 500 HGT-1 and 250 HGT-1 + 250 CAF spheroids formation going from 0 to 8 days of culture, captured with the Incucyte live imaging microscope.
